# Effects of *PHENYLALANINE AMMONIA LYASE* (*PAL*) knockdown on cell wall composition, biomass digestibility, and biotic and abiotic stress responses in *Brachypodium*

**DOI:** 10.1093/jxb/erv269

**Published:** 2015-06-19

**Authors:** Cynthia L. Cass, Antoine Peraldi, Patrick F. Dowd, Yaseen Mottiar, Nicholas Santoro, Steven D. Karlen, Yury V. Bukhman, Cliff E. Foster, Nick Thrower, Laura C. Bruno, Oleg V. Moskvin, Eric T. Johnson, Megan E. Willhoit, Megha Phutane, John Ralph, Shawn D. Mansfield, Paul Nicholson, John C. Sedbrook

**Affiliations:** ^1^School of Biological Sciences, Illinois State University, Normal, IL 61790USA; ^2^US Department of Energy Great Lakes Bioenergy Research Center, Madison, WI 53706, USA; ^3^Department of Crop Genetics, John Innes Centre, Norwich Research Park, Norwich NR4 7UH, UK; ^4^USDA, Agricultural Research Service, National Center for Agricultural Utilization Research, Crop Bioprotection Research Unit, Peoria, IL 61604, USA; ^5^Department of Wood Science, University of British Columbia, Vancouver, V6T 1Z4, Canada; ^6^US Department of Energy Great Lakes Bioenergy Research Center, Michigan State University, East Lansing, MI 48824, USA; ^7^Department of Biochemistry, Wisconsin Energy Institute, University of Wisconsin, Madison, WI 53706, USA

**Keywords:** Bioenergy, ferulic acid, *Fusarium*, grass, herbivory, lignin, phenylpropanoid, saccharification, tyrosine ammonia lyase, ultraviolet light.

## Abstract

The phenylpropanoid pathway in plants synthesizes a variety of structural and defence compounds, and is an important target in efforts to reduce cell wall lignin for improved biomass conversion to biofuels. Little is known concerning the trade-offs in grasses when perturbing the function of the first gene family in the pathway, *PHENYLALANINE AMMONIA LYASE* (*PAL*). Therefore, *PAL* isoforms in the model grass *Brachypodium distachyon* were targeted, by RNA interference (RNAi), and large reductions (up to 85%) in stem tissue transcript abundance for two of the eight putative *BdPAL* genes were identified. The cell walls of stems of *BdPAL*-knockdown plants had reductions of 43% in lignin and 57% in cell wall-bound ferulate, and a nearly 2-fold increase in the amounts of polysaccharide-derived carbohydrates released by thermochemical and hydrolytic enzymic partial digestion. *PAL*-knockdown plants exhibited delayed development and reduced root growth, along with increased susceptibilities to the fungal pathogens *Fusarium culmorum* and *Magnaporthe oryzae*. Surprisingly, these plants generally had wild-type (WT) resistances to caterpillar herbivory, drought, and ultraviolet light. RNA sequencing analyses revealed that the expression of genes associated with stress responses including ethylene biosynthesis and signalling were significantly altered in *PAL* knocked-down plants under non-challenging conditions. These data reveal that, although an attenuation of the phenylpropanoid pathway increases carbohydrate availability for biofuel, it can adversely affect plant growth and disease resistance to fungal pathogens. The data identify notable differences between the stress responses of these monocot *pal* mutants versus *Arabidopsis* (a dicot) *pal* mutants and provide insights into the challenges that may arise when deploying phenylpropanoid pathway-altered bioenergy crops.

## Highlight

Reducing the function of PAL, the first enzyme in the phenylpropanoid pathway, in *Brachypodium distachyon* alters cell wall composition, increases fungal susceptibility, but minimally affects caterpillar herbivory and abiotic stress tolerance.

## Introduction

Lignocellulosic biomass from grasses is an attractive feedstock for liquid biofuels and electricity generation. It is estimated that grasses could contribute more than half of the 1 billion dry tons of biomass envisaged for biofuel production in the USA each year (US Department of Energy, 2011).

Lignocellulosic biomass consists primarily of thick secondary cell walls present in the vasculature and structural support tissues of plant stems and leaves. Secondary cell walls are composed of the polysaccharides cellulose and hemicelluloses, the latter cross-linked to and imbedded within lignin (Carpita and McCann, 2002). Lignin is a heterogeneous phenolic polymer composed primarily of guaiacyl (G) and syringyl (S) units, along with lesser amounts of *p*-hydroxyphenyl (H) units, interconnected by β-*O*-4-aryl ether bonds and other covalent linkages (Freudenberg and Neish, 1968; Ralph *et al.*, 2004*b*). Unique to commelinid monocotyledons are *p*-coumarate (*p*CA) and ferulate (FA) units that decorate lignin and arabinoxylan (a hemicellulose) as ester-linked appendages (Mueller-Harvey *et al.*, 1986; Lam *et al.*, 1992; Petrik *et al.*, 2014). FA is acylated to arabinosyl units that get incorporated into hemicelluloses; oxidative coupling of FA in the cell wall forms cross-links between hemicellulose polymers as well as between hemicellulose and lignin (Ralph *et al.*, 2004*a*; de O Buanafina, 2009; Ralph, 2010). Although the function of *p*CA appendages is not entirely clear (their toxicity might deter microbes, and they can aid in syringyl unit polymerization; Hatfield *et al.*, 2008; Ralph, 2010), FA cross-linking provides structural strength to the cell wall and is postulated to form a physical and toxic barrier to pests and pathogens (Bily *et al.*, 2003; Ralph *et al.*, 2008; Barros-Rios *et al.*, 2011; de O Buanafina and Fescemyer, 2012).

The hydrophobicity of lignin facilitates solute transport through the vasculature and limits plant water loss during evapotranspirative processes. The properties that make lignin effective against biotic and abiotic challenges, however, limit lignocellulosic biomass digestibility to ruminant livestock as well as rendering it more recalcitrant to conversion to liquid biofuels (Jung and Allen, 1995). Studies have shown that reducing lignin content in plants significantly lowers biomass recalcitrance and can improve biofuel conversion efficiencies. However, these gains can be offset by losses in biomass yield and/or plant fitness (Jackson *et al.*, 2008; Dien *et al.*, 2011; Fu *et al.*, 2011; Mansfield *et al.*, 2012; Bonawitz and Chapple, 2013).

The monolignols sinapyl, coniferyl, and *p*-coumaryl alcohol, that become oxidatively coupled in the cell wall to form the S, G, and H units in lignin, respectively, are synthesized via the phenylpropanoid pathway (Boerjan *et al.*, 2003). Offshoots of the phenylpropanoid pathway give rise to a variety of phenolic secondary metabolites, including flavonoids, coumarins, and lignans, that have diverse functions such as protecting against photo-oxidative damage, participating in developmental and stress signalling, mediating plant–microbe interactions, and defending against pathogens and pests (Lattanzio *et al.*, 2006; Treutter, 2006; Johnson *et al.*, 2008; Naoumkina *et al.*, 2010; Fang *et al.*, 2013). Phenylalanine ammonia lyase (PAL) and its conversion of l-phenylalanine to *trans*-cinnamate and ammonia is the first committed step in the phenylpropanoid pathway, redirecting large amounts of fixed carbon from primary to secondary metabolism.

Although much has been learned about lignin biosynthesis in dicotyledonous (dicot) plant species, gaps remain in our understanding of how the phenylpropanoid pathway is wired in monocotyledonous (monocot) plants, including grasses, due to a paucity of mutants (Gray *et al.*, 2012). Studies suggest that differences exist. For example, a subset of the PAL proteins in monocots are uniquely bifunctional in that they can utilize as substrates both phenylalanine and tyrosine (Rösler *et al.*, 1997; Watts *et al.*, 2006); that is, these particular PAL isoforms also have tyrosine ammonia lyase (TAL) activity, possibly directing l-tyrosine into the pathway by producing the intermediate *p*-coumarate. In addition, monocot pathways uniquely harbour known and unknown transferases thought to funnel considerable amounts of the phenylpropanoid pathway intermediates *p*-coumaroyl-CoA and feruloyl-CoA toward the production of the aforementioned hydroxycinnamate appendages *p*CA and FA on both polysaccharides and lignin (Petrik *et al.*, 2014).

Genetically altering the phenylpropanoid pathway is viewed as an attractive route to improving biomass deconstruction for generation of biofuels by reducing lignin levels or altering its deconstructive properties. Much remains to be learned, however, with respect to how such manipulations affect plant growth and susceptibilities to abiotic and biotic challenges. To improve understanding of how the first of the pathway genes is involved in grasses, *PAL* RNA interference (RNAi) mutant lines were generated in the grass model *Brachypodium distachyon* and characterized. It was found that reducing *PAL* activity provided the desired benefit of substantially reducing lignin and increasing stem biomass digestibility. However, trade-offs were observed. *PAL* knockdown plants grew more slowly than the wild type (WT) and exhibited increased susceptibility to fungal pathogens, but generally exhibited WT resistances to caterpillar herbivory, drought, and UV light challenges, suggesting that reducing lignin in grasses may result in a minimal trade-off with respect to these responses.

## Materials and methods

### Plant transformation and growth, qRT–PCR, and RNA-seq

See the Supplementary Materials and methods.

### Digestibility assays

See the Supplementary Materials and methods.

### PAL and TAL activity assays

Protein extractions and kinetic assays were performed similarly to as described by Cheng and Breen (1991) and Rösler *et al.* (1997). Pooled first internode culm plus leaf sheath tissue (0.2g) from 35–43-day-old soil-grown plants of the same developmental stage, as well as the distal 4cm of roots from 7- to 10-day-old seedlings grown on agar-containing growth medium, was flash-frozen in liquid nitrogen and stored at –80 °C. Frozen tissue was ground at 30 Hz for 45 s in a Qiagen TissueLyser with the aid of three pre-chilled 5mm 440C stainless ball bearings. Pulverized tissue was washed with 1ml of ice-cold acetone, incubated at –20 °C for 15min, and centrifuged at 16 000 *g* for 15min at 4 °C. The pellets were air-dried under nitrogen on ice, then extracted by gentle rotation at 4 °C in 100mM sodium borate pH 8.8/2mM EDTA containing 5mM 2-mercaptoethanol added just before use at 5ml g^–1^ fresh weight. After 1h, samples were centrifuged as above and the supernatant was used as plant extract in kinetic assays. Protein quantitation of plant extracts was performed using the BCA assay as per the manufacturer’s instructions (Pierce). Substrates l-phenylalanine and l-tyrosine, and products *trans*-cinnamic acid and *p*-coumaric acid for use as standards, were obtained from Sigma-Aldrich.

PAL activity was quantified by the production of *trans*-cinnamic acid, monitored by taking absorption spectra at 290nm (Zucker, 1965; Rösler *et al.*, 1997) every minute for 20min at 37 °C, using a NanoDrop 2000c spectrophotometer (Thermo Scientific). The assay mixture contained 61mM l-phenylalanine, 30mM sodium borate buffer (pH 8.8), and 75 μl of plant extract, in a 1ml reaction volume. The substrate was added after a 10min pre-incubation period at 37 °C. Plant extract pre-incubated in buffer without substrate was used as the blank before each assay. Each sample was analysed in duplicate. Under the experimental conditions, the increase in absorbance at 290nm was linear for at least 2h. TAL activity was determined in a similar manner by monitoring the production of *p*-coumaric acid at 310nm (Rösler *et al.*, 1997). The 1ml assay mixture contained 1.9mM l-tyrosine, 30mM sodium borate buffer (pH 8.8), and 75 μl of plant extract. The temperature and blanking protocol were identical to those in the PAL assay.

### Stem sectioning and phloroglucinol staining

Hand-cut stem cross-sections from plants 35–40 d after planting were stained with phloroglucinol as described in Petrik *et al.* (2014).

### Cell wall compositional analyses

Stem samples were cut into ~1cm pieces and then ground in a Wiley mill to pass a 40-mesh sieve. The ground tissue was then wrapped in folded filter paper envelopes and placed in a Soxhlet extractor to remove extractives by overnight acetone extraction. Following extraction, samples were dried overnight at 50 °C. All samples were processed in triplicate using 3×0.2g. To avoid any experimental bias during processing, random sample IDs were assigned to each sample.

#### Acid hydrolysis 

For each sample, the exact dry weight measured into a serum bottle was recorded (~0.1g) and 3ml of cold 72% sulphuric acid was added. Each sample was mixed every 10min for 2h, at which point 112ml of deionized water was added. The bottles were capped, sealed, and autoclaved for 1h at 121 °C. Pre-weighed oven-dried sintered-glass crucibles (medium coarseness, Pyrex) were used to filter the acid-insoluble residue. A 10ml sample of the filtered hydrolysate was retained and stored at 4 °C for use in quantifying the acid-soluble lignin and the composition of sugars.

The acid-insoluble residue retained on the sintered-glass crucibles was washed with 150ml of warm H_2_O. After oven-drying overnight, the weight of the residue was measured to determine the amount of acid-insoluble lignin. The acid-soluble lignin was measured using the absorbance at 205nm of a 1:3 dilution of the hydrolysate. An extinction coefficient of 110 l g^–1^ cm^–1^ was used in the Beer–Lambert calculations.

#### Structural carbohydrates 

Acid-hydrolysed sugars were analysed by high-performance liquid chromatography (HPLC). Neutral sugars were separated with a CarboPac PA1 column using the pulsed amperometric detection mode with a Dionex HPLC apparatus. A 15 μl volume of filtered sample was injected on to the column and a water eluent was used at a flow rate of 0.8ml min^–1^ to separate the sugars. A column clean-up was performed using 250mM NaOH between samples. Calibration curves were prepared using solutions of sugar standards run in parallel through acid hydrolysis. The amounts of fucose, arabinose, rhamnose, galactose, glucose, xylose, and mannose were evaluated using these calibration curves and by taking into account the water added during hydrolysis of glycan chains.

#### Thioacidolysis method 

Lignin monomer ratios were estimated using the rapid, small-scale thioacidolysis procedure, according to Robinson and Mansfield (2009).

#### Cell wall crystallinity 

Cell wall crystallinity was determined by X-ray diffraction on a D-8 Discover X-ray diffractometer equipped with a GADDS detector, as described in Coleman *et al.* (2009).

#### FA and *p*CA determination 

Saponification and gas chromatography-flame ioization detection (GC-FID) to quantify total esterified *p*CA and FA was performed as described in Petrik *et al.* (2014).

### Fungal bioassays

#### *Brachypodium* growth conditions 

Plants used for spray infection tests were grown under 16h/8h light–dark at 22 °C as reported previously (Peraldi *et al.*, 2011), except for infection with *Magnaporthe oryzae* where plants were grown at 24 °C. Seedlings employed for root infection were grown for 1 d at 22 °C under a 16h/8h light–dark photoperiod before transferring onto filter paper (Sartorius; grade 292) placed on the surface of 0.8% water agar in 10cm square clear plastic plates. Plates were placed upright at about a 60 ° angle for 2–3 d under the same conditions to encourage uniform root growth down the plate.

In experiments to examine the effect of ethylene on root growth, seedlings were germinated and grown as described above before transfer to plates containing 0.8% water agar amended with either the ethylene precursor 1-aminocyclopropane-1-carboxylic acid (ACC), or the inhibitor of ethylene biosynthesis aminoethoxyvinylglycine (AVG). To test the effect of ACC treatment on Bd21-3 and *BdPAL* RNAi1 line 1 root growth, seedlings were transferred on 0.8% agar media containing 0, 0.25, 0.5, 0.75, and 1mM ACC after 1 d of growth on control agar plates. Similarly, to test the effect of AVG, seedlings were transferred onto 0.8% agar media containing 0, 0.5, and 2 μM AVG after 1 d of growth on control agar plates.

#### Fungal material and inoculum 

Deoxynivalenol- (DON) producing *Fusarium culmorum* isolate Fu42 from the culture collection of the John Innes Centre was maintained and conidial inoculum prepared as detailed by Peraldi *et al.* (2011). *Magnaporthe oryzae* wheat-adapted isolate BR32 (kindly provided by Dr G.R.D. McGrann, John Innes Centre, Norwich, UK) was maintained at 24 °C as detailed by Tufan *et al.* (2009).

#### Magnaporthe infection test 

Conidia were washed from plates of 10- to 14-day-old *M. oryzae* colonies with sterile distilled water (SDW). The conidial suspension was adjusted to 1×10^5^ conidia ml^–1^, amended with 0.01% Tween-20, and used to spray plants until run-off. Plants were kept under a plastic cover previously misted with SDW to increase relative humidity until disease assessment, 5 d following inoculation.

#### *Fusarium* infection tests 

Two independent detached leaf infection experiments were performed and analysed as described by Peraldi *et al.* (2011). Two independent *Fusarium* root infection tests were performed, in addition to one including investigation of the effects of ACC on susceptibility. For the first test, eight replicate plates were used and three seedlings each of Bd21-3 and *BdPAL* RNAi1 line 1 were grown in each plate to allow direct comparison between the lines. Root tips were inoculated with *F. culmorum* slurry (2ml) of homogenized 2-week-old colonies grown on V8 vegetable juice amended with potato dextrose agarose (PDA). At 4 d post-inoculation, the slurry was delicately washed off. Roots were photographed using a Panasonic DMC-FZ18 digital camera and lesions measured using the ImageJ software (Abramoff *et al.*, 2004). To examine the effect of ethylene on susceptibility, seedlings were germinated and grown for 3 d as described above on filter paper laid over 0.8% water agar to produce sufficient root growth for the bioassay. After this time, the filter paper, with the seedlings attached, was removed and transferred to plates containing 0.8% water agar amended with ACC, or AVG, or unamended agar as a control. Seedlings were left to recover and absorb the chemical tested for 6h for the ACC treatment and 24h for the AVG treatment, then root tips were inoculated as above.

### Insect bioassays

#### Insects 

Fall armyworms and another grass/maize pest, the corn earworm (*Helicoverpa zea*), were reared on a pinto bean diet at 27±1 °C, 50±10% relative humidity, and a 14:10 light:dark photoperiod as previously described (Dowd, 1988). First instar larvae were used for assays.

#### Bioassays 

Leaf and stem sections were obtained from *BdPAL* RNAi1 line 1 plants that had begun to form flower heads, but had not yet flowered. Leaf and stem sections from the same position relative to the terminal leaf of each plant were removed from plants and caged with insects as described previously (Johnson and Dowd, 2004; Dowd *et al.*, 2007). Leaf or stem sections were placed in Petri dishes with tight-fitting lids, containing moistened filter paper, along with 10 first instar larvae. Dishes were examined for feeding levels and survivorship of larvae for 2 d, and then assays were frozen. Due to their small size, larvae from each dish were weighed as a group using a Mettler Model AX105 analytical balance.

### Statistical analyses

Significant differences in *BdPAL1* and *BdPAL2* gene expression and digestibility (free glucose amount subtracted from grinding alone, hot water, and sulphuric acid pre-treatments), were determined by analysis of variance (ANOVA) using SAS v 9.3 Proc GLM. Biomass traits including hemicellulose composition, cell wall crystallinity, Klason lignin amounts, *p*CA and FA amounts, as well as leaf-rolling and UV damage indices were analysed by Student’s *t*-tests in SAS v. 8.0 or higher or in MS Excel. Significant differences in insect mortality or frequency of stem feeding were determined by χ^2^ analysis using SAS Proc Freq. Insect feeding assay ratings and weights were determined by SAS Proc GLM. Significant correlations between insect feeding ratings and mortality were determined using SAS Proc Reg. All root growth and disease symptom data were analysed using a generalized linear model (GLM) in the software package GENSTAT v16.0 (Lawes Agricultural Trust, Rothamsted Experimental Station, UK). Unpaired *t*-tests calculated within the GLMs were used to assess differences in growth response and disease symptom severity between Bd21-3 and *BdPAL* RNAi1 line 1 lines.

## Results

### The *Brachypodium* genome encodes eight putative *PAL* genes

The genomes of both dicot and monocot plant species harbour multiple *PAL* genes. For example, the *Arabidopsis* genome contains four *PAL* genes (Rawal *et al.*, 2013), whereas the rice genome appears to harbour nine (Supplementary Fig. S1 available at *JXB* online). To identify the putative *PAL* genes in *B. distachyon*, BLAST searches of the annotated *Brachypodium* genome (International Brachypodium Initiative, 2010) were performed using known PAL sequences (Minami *et al.*, 1989; Huang *et al.*, 2010) as queries. Eight candidate *BdPAL* genes were identified and predicted to encode proteins sharing from 75.3% to 98.5% amino acid sequence identity with each other (Table 1; Supplementary Fig. S1). Notably, Bradi3g49250 stands out as relatively more diverged compared with the other putative BdPAL proteins.

**Table 1.** Percentage amino acid sequence identities of the eight predicted Brachypodium BdPAL proteins, as determined by Clustal W analysis

Divergence	Percentage identity									
	1	2	3	4	5	6	7	8		
1		75.6	76.0	76.6	76.3	75.7	75.7	75.3	1	BdPAL1 Bradi3g49250
2	29.5		86.1	86.4	86.5	85.8	85.0	87.4	2	BdPAL2 Bradi3g49260
3	29.0	15.4		94.2	93.5	93.8	93.0	87.6	3	BdPAL3 Bradi3g49270
4	28.1	15.1	6.0		98.5	93.7	92.8	88.2	4	BdPAL4 Bradi3g49280
5	28.6	14.9	6.8	1.6		93.0	92.1	88.3	5	BdPAL5 Bradi3g48840
6	29.4	15.8	6.5	6.6	7.4		98.7	86.8	6	BdPAL6 Bradi3g47110
7	29.4	16.8	7.4	7.5	8.3	1.3		86.3	7	BdPAL7 Bradi3g47120
8	30.0	13.8	13.6	12.9	12.7	14.6	15.2		8	BdPAL8 Bradi3g15830
	1	2	3	4	5	6	7	8		

A comparison of these sequences with those of 195 divergent PAL proteins (Calabrese *et al.*, 2004) revealed that all eight possessed the highly conserved alanine–serine–glycine triad (Supplementary Fig. S2 at *JXB* online) that forms the highly electrophilic prosthetic group methylidene-imidazolone (MIO) necessary for ammonia lyase catalytic activity (Langer *et al.*, 2001; MacDonald and D’Cunha, 2007). Moreover, the Bradi3g49250 polypeptide was uniquely predicted to have a histidine residue at position 123 (His123); the other putative *Brachypodium* PAL polypeptides harbour a phenylalanine residue at that position (see consensus position 149 in Supplementary Fig. S2). Watts *et al.* (2006) showed that this residue position is key in determining substrate specificity; PAL proteins harbouring phenylalanine at this active site specifically utilize phenylalanine as a substrate, whereas plant PAL proteins having a histidine at this position are able to utilize both tyrosine and phenylalanine. It is therefore hypothesized that Bradi3g49250 encodes a protein having both PAL and TAL activities.

**Fig. 1.**
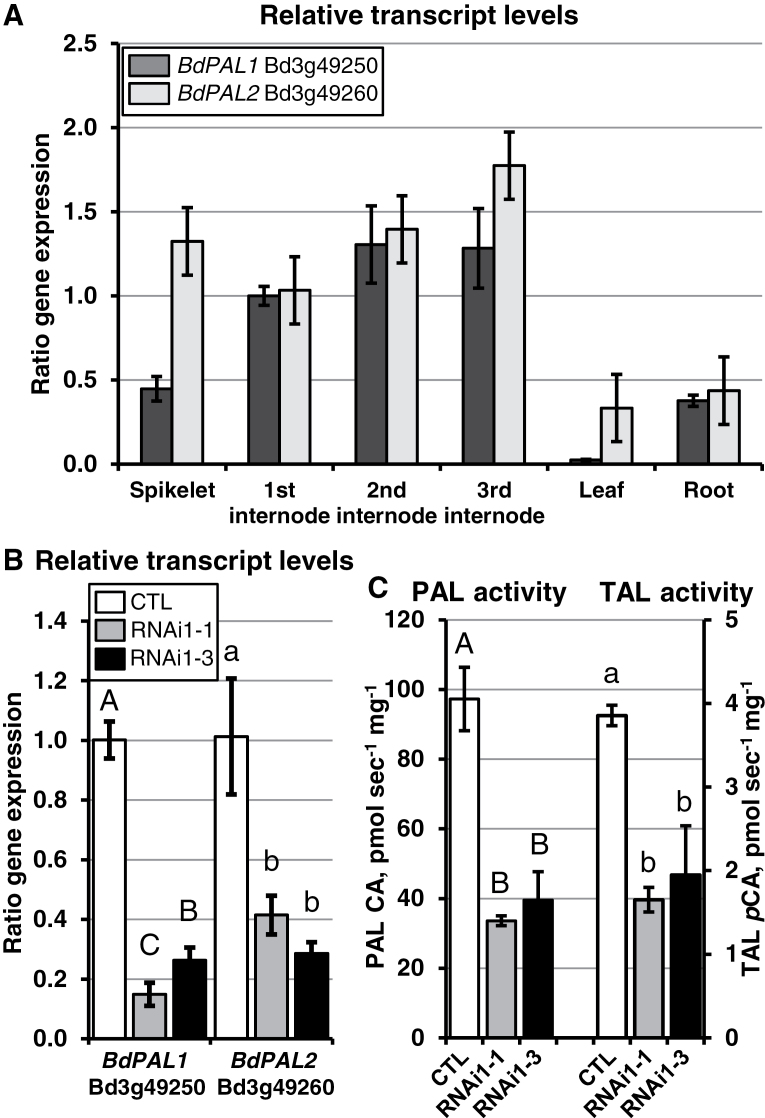
Bradi3g49250 (*BdPAL1*) and Bradi3g49260 (*BdPAL2*) transcript levels in *Brachypodium* CTL and *BdPAL* RNAi mutant tissues, and related protein activity. (A) Relative amounts of *BdPAL1* and *BdPAL2* transcripts in various CTL plant tissues, as determined by qRT–PCR. (B) *BdPAL1* and *BdPAL2* transcript levels in *BdPAL* RNA1-1 and 1-3 third internode stem tissues compared with the CTL. (C) Amounts of PAL activity (left three columns) and TAL activity (right three columns) in culm first internode extracts. Note that PAL activity is ~25-fold higher than TAL activity. In (A), means (represented by columns) were normalized to the first internode mean transcript level value, which was set to 1, using expression of *BdUBC18* (Bradi4g00660) as reference. Bars represent standard deviations (SDs). Different letters above columns represent significant differences. *n*=4 biological reps and *n*=3 technical reps for (A) and (B), and 2 and 2 for (C).

RNA sequencing (RNA-seq) analyses were performed to determine the relative expression levels of the eight putative *BdPAL* genes in stem plus leaf sheath tissues. As shown in Table 2, Bradi3g49250 and Bradi3g49260 were found to have transcript levels at least 24-fold higher than that of the next most highly expressed *BdPAL* gene, Bradi3g49280. Bradi3g49280 and Bradi5g15830 had modest transcript abundances in these tissues, whereas transcripts attributed to Bradi3g49270, Bradi3g48840, Bradi3g47110, and Bradi3g47120 were low or undetected. Quantitative reverse transcription–PCR (qRT–PCR) analysis was performed to determine relative expression levels of Bradi3g49250 and Bradi3g49260 across various tissues, revealing that these two genes were expressed in all tissues examined, with both being expressed at relatively high levels in stem internodes at all developmental stages (Fig. 1A). Bradi3g49250 transcript abundances were relatively low in leaf tissues, suggesting that Bradi3g49260 might play a more prominent role in leaves.

**Table 2.** Comparison of RNA-seq-derived average transcript levels of putative BdPAL genes in BdPAL RNAi1-1 stem plus leaf sheath tissues versus empty vector control (CTL)

Locus ID	Gene ID	Gene name	CTL	*BdPAL* RNAi1-1	Fold change	*P*-value	Adjusted *P*-value
LOC100839236	Bradi3g49250	*BdPAL1*	20 340	8902	0.4	6.7E-08	5.4E-05
LOC100840340	Bradi3g49260	*BdPAL2*	11 172	6829	0.6	3.7E-08	3.4E-05
LOC100841149	Bradi3g49270	*BdPAL3*	10	7	0.7	0.695	1
LOC100841454	Bradi3g49280	*BdPAL4*	475	658	1.4	0.008	0.383
LOC100829928	Bradi3g48840	*BdPAL5*	64	70	1.1	0.749	1
LOC100843583	Bradi3g47110	*BdPAL6*	0	1	NA	0.997	1
LOC100844192	Bradi3g47120	*BdPAL7*	0	0	NA	0.991	1
LOC100845653	Bradi5g15830	*BdPAL8*	370	377	1.0	0.991	1

*n*=3 for RNAi1-1 and CTL samples each.

Mutwil *et al.* (2011) recently developed a web-based platform called PlaNet that deciphers and predicts transcriptomic co-expression networks based on publicly available microarray data from a variety of plant species including *B. distachyon* (http://aranet.mpimp-golm.mpg.de/). PlaNet determined with high confidence that Bradi3g49250 and Bradi3g49260 were temporally and spatially co-expressed with each other and with known and predicted *Brachypodium* monolignol biosynthetic genes including *CAFFEIC ACID O-METHYL-TRANSFERASE* (*COMT*; Bradi3g16530; Dalmais *et al.*, 2013; Trabucco *et al.*, 2013; Wu *et al.*, 2013), *CINNAMYL ALCOHOL DEHYDROGENASE* (*CAD*; Bradi3g06480; Bukh *et al.*, 2012; Bouvier d’Yvoire *et al.*, 2013), *p-COUMAROYL-CoA:MONOLIGNOL TRANSFERASE* (*PMT*; Bradi2g36910; Petrik *et al.*, 2014), and *CINNAMOYL-CoA REDUCTASE* (*CCR*; Bradi3g36887) (Supplementary Tables S1, S2 at *JXB* online). Bradi3g49250 and Bradi3g49260 were also identified as being co-expressed with genes putatively involved in phenylpropanoid secondary metabolism, cellulose and cell wall biosynthesis, and biotic and abiotic stress responses (Supplementary Tables S1, S2). None of the other six putative *BdPAL* genes was identifed as being co-expressed with these two genes or with other monolignol biosynthetic genes. They might therefore be involved in the biosynthesis of phenolic secondary metabolites other than monolignols, or in lignin formation in other tissues or organs not represented by the microarray data sets, and/or may be induced by abiotic and/or biotic challenges to form defence lignins.

Based on the above-noted expression and sequence data and the fact that greatly reduced Bradi3g49250 and Bradi3g49260 transcript levels in plants harbouring a *BdPAL* RNAi construct co-segregated with predicted *PAL* loss-of-function phenotypes (described below), Bradi3g49250 is here designated as *BdPAL1* and Bradi3g49260 as *BdPAL2*. To accommodate scientific discussions, names for the other *Brachypodium PAL*-like genes are also here designated: *BdPAL3*, Bd3g49270; *BdPAL4*, Bd3g49280; *BdPAL5*, Bd3g48840; *BdPAL6*, Bd3g47110; *BdPAL7*, Bd3g47120; and *BdPAL8*, Bd5g15830. The order of the gene names was chosen based on protein relatedness as determined by Clustal W analysis as well as chromosomal linkages between loci. For example, the BdPAL3–BdPAL7 protein sequences cluster together phylogenetically (Supplementary Fig. S1 at *JXB* online), whereas the *BdPAL1*–*BdPAL4* loci are physically adjacent to each other on chromosome 3, as are *BdPAL6* and *BdPAL7*.

Mutational analyses have shown that the four *PAL* genes encoded by the *Arabidopsis thaliana* genome function redundantly (Rohde *et al.*, 2004; Huang *et al.*, 2010). To overcome possible *PAL* gene functional redundancies in *Brachypodium*, a *BdPAL* RNAi hairpin construct was designed to knock down expression of multiple *BdPAL* genes. This RNAi construct consisted of a 619 nucleotide long fragment of the Bd3g49260-derived cDNA, the sequences sharing 100% identity with stretches of 21 nucleotides or longer present in all seven of the other *BdPAL* open reading frames (ORFs; Supplementary Fig. S3 at *JXB* online). Given that the RNAi cellular machinery recognizes 21 nucleotide long sequences for gene silencing (Watson *et al.*, 2005), this RNAi construct could potentially target transcript degradation of multiple *BdPAL* genes.

### *BdPAL* RNAi plants have greatly reduced expression of the two highest stem-expressed *BdPAL* genes, reduced PAL and TAL activity, and reduced lignin phenotypes

**Fig. 2.**
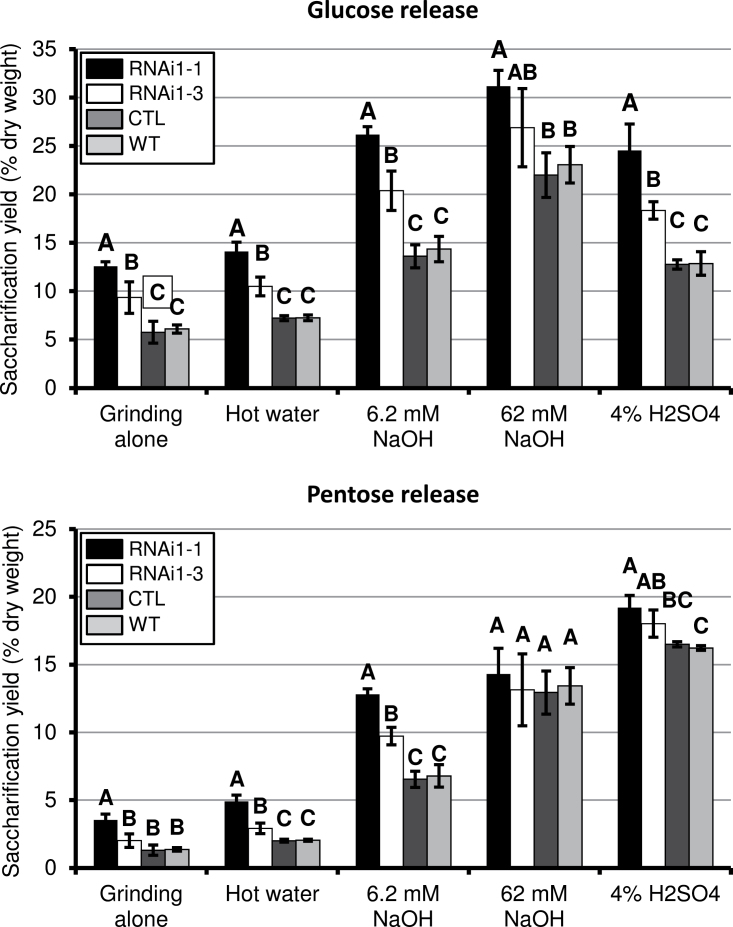
Digestibility of stem biomass, comparing *BdPAL* RNAi1-1 and 1-3 with empty vector control (CTL) and the WT. Shown are the average amounts of glucose (top chart) and pentose sugars (bottom chart) released per milligram of senesced ground biomass using the various pre-treatments along with partial enzyme hydrolysis. Vertical bars represent the SD. Different letters for a given treatment represent significant differences. *n*=5 biological reps, *n*=3 technical reps.

**Fig. 3.**
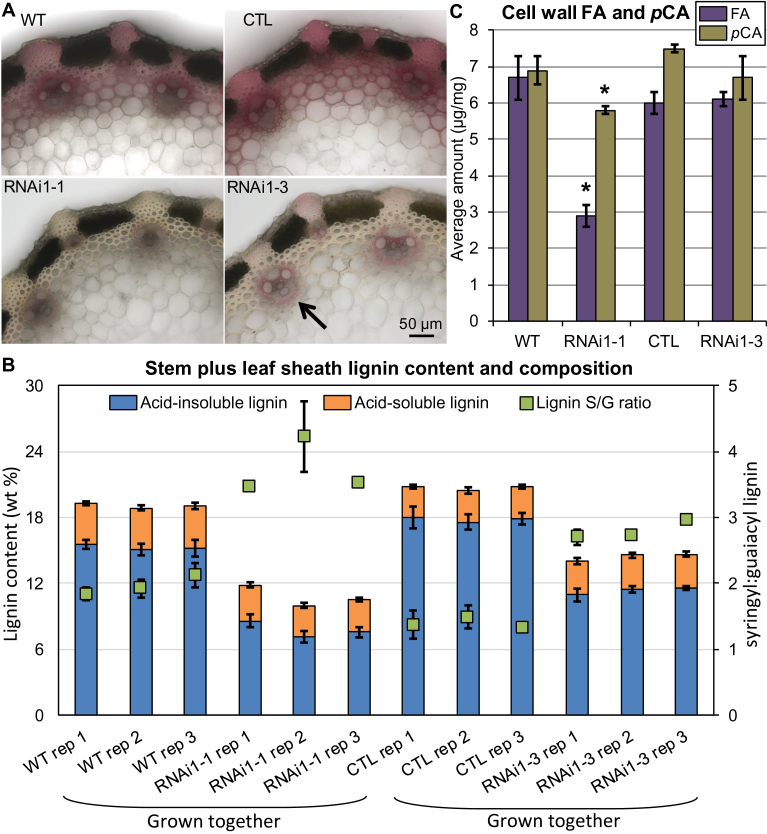
*BdPAL* RNAi cell walls have altered lignin composition and reduced FA and *p*CA content. (A) Phloroglucinol staining of stem sections. Lighter staining is consistent with less lignin. Note that vascular bundles (marked by an arrow) appear normal (e.g. no collapsed cells) in both RNAi lines. (B) Amounts of acid-insoluble and acid-soluble lignin, as well as lignin S:G units ratios, in senesced stems plus leaf sheaths. (C) The amounts of ferulate (FA) and *p*-coumarate (*p*CA) released from lignin (*p*CA) and hemicelluloses (FA and *p*CA) upon 2M NaOH treatment. Vertical bars represent the SD. For (B), each column represents a biological rep with three technical reps averaged; significant differences can be found in Supplementary Table S3 at *JXB* online. For (C), *n*=3 biological reps and *n*=2 technical reps. Asterisks denote Student’s *t*-test significant differences (*P*<0.01) between each *PAL* knocked-down line and its corresponding control grown concurrently (*BdPAL* RNAi1-1 versus WT; *BdPAL* RNAi1-3 versus CTL).

*Brachypodium* transformants harbouring the *BdPAL* RNAi construct were generated and screened for lignin-related phenotypes. It was hypothesized that the stem biomass of reduced-lignin plants would stain a lighter red with the phloroglucinol lignin stain and would release relatively larger amounts of cell wall polysaccharide-derived sugars upon saccharification, compared with the WT. This was, in fact, observed in plants from seven independent transgenic lines, with the relative strengths of these phenotypes mirroring the relative amounts of *BdPAL* gene knockdown (Figs 1B, 2, 3, and data not shown). T_2_ generation plants from two independent lines (*BdPAL* RNAi1 lines 1 and 3) were characterized in detail and found to have stem biomass with substantially higher digestibility compared with that of both WT and empty vector control (CTL) plants (Fig. 2). A more encompassing evaluation of biomass digestibility showed that stem biomass from both lines had significantly higher release of glucose and pentose sugars associated with a variety of chemical pre-treatments including hot water, weak acid, and weak base followed by partial enzymatic hydrolysis. With all pre-treatments, *BdPAL* RNAi1 line 1 senesced stem biomass had the greatest amounts of sugars released, in most cases being twice the amount released from the WT (Fig. 2) under the chosen assay (Santoro *et al.*, 2010).

The high digestibility phenotypes of *BdPAL* RNAi1 lines 1 and 3 (hereafter referred to as RNAi1-1 and RNAi1-3) plants were found to be heritable, as were related phenotypes (described below). Moreover, co-segregation analyses showed the following assessed phenotypes to have 100% co-segregation with the RNAi construct in the T_2_ generation segregating populations (significance determined using Fisher’s exact test): reduced phloroglucinol staining of stem sections (RNAi1-1, *P*=5.8×10^–4^, *n*=13; RNAi1-3, *P*=0.0286, *n*=7); lignin monomer ratios as determined by the thioacidolysis method (RNAi1-3, *P*=0.0286, *n*=7); and blackened leaf collar phenotype (RNAi1-1, *P*=4.2×10^–6^, *n*=33; RNAi1-3, *P*=1.3×10^–3^, *n*=12). Phloroglucinol (Wiesner stain) is an organic dye that labels (hydroxy)cinnamaldehyde end groups (Fig. 3A) (Lin and Dence, 1992; Kim *et al.*, 2002).

To determine which *BdPAL* genes in *BdPAL* RNAi1 plants had reduced transcript levels as a consequence of the RNAi construct, total RNA was isolated from stem plus leaf sheath tissues of flowering plants and analysed by RNA-seq and qRT–PCR. It was hypothesized that the expression levels of Bradi3g49260 and perhaps Bd3g49250 would be reduced given that (i) the RNAi construct was derived from Bradi3g49260 sequences; (ii) these two putative *BdPAL* genes are by far the most highly expressed in stem tissues; and (iii) both were found to be co-expressed with known monolignol biosynthetic genes (discussed above). The data in Table 2 show that Bd3g49250 and Bd3g49260 transcripts were both substantially reduced in *BdPAL* RNAi1-1 stem tissues compared with empty vector control (CTL) as determined by RNA-seq analysis. On the other hand, transcript levels of the modestly expressed Bd3g49280 gene appeared to be increased (1.4-fold higher compared with CTL; *P*=0.008, adjusted *P* not significant), whereas transcript levels corresponding to the remaining five *BdPAL* genes were indistinguishable from the CTL. Moreover, qRT–PCR analyses showed that average Bd3g49250 transcript levels were reduced by 85% and 74% in RNAi1-1 and 1-3 plants, respectively, whereas average Bd3g49260 transcript levels were reduced by 59% and 71% in RNAi1-1 and 1-3 plants, respectively, compared with CTL (Fig. 1B).

To determine if PAL enzymatic activity reductions mirrored the *BdPAL* transcript level reductions, crude protein extracts were prepared from the first internode of developing culms and from roots, then the conversion of l-phenylalanine to *trans*-cinnamate was assayed by tracking UV light absorbance increases attributable to *trans*-cinnamic acid accumulation. Comparing these measurements with a *trans*-cinnamic acid standard curve, it was determined that *BdPAL* RNAi1-1 and 1-3 stem extracts had 65% and 59% reductions in PAL activity compared with CTL stem extracts, respectively, which is as expected based on the transcript level reductions (Fig. 1C). Moreover, *BdPAL* RNAi1-1 root extracts had, on average, a 62% reduction in PAL activity compared with the WT (Supplementary Fig. S4 at *JXB* online). Given that the Bd3g49250 predicted polypeptide sequence suggests that it could also have TAL activity (discussed above), an assay was conducted to determine conversion of l-tyrosine to *p*-coumaric acid by tracking *p*-coumaric acid-attributable absorbance at 310nm (Rösler *et al.*, 1997). As hypothesized, a substantially slower increase in absorbance was detected in the *BdPAL* RNAi1-1 and 1-3 stem extracts compared with the WT. Comparing the absorbance with that of a *p*-coumaric acid standard curve, 57% and 50% reductions in TAL activity were estimated for *BdPAL* RNAi1-1 and 1-3 stem extracts, respectively, compared with the WT (Fig. 1C). TAL activity in WT root extracts was low, making it difficult to discern whether activity was reduced in *BdPAL* RNAi1-1 roots (Supplementary Fig. S4).

In order to obtain a more accurate determination of the degrees to which lignin was reduced in T_3_ *BdPAL1* RNAi1-1 and RNAi1-3 plants, senesced stem plus leaf sheath tissues were assessed for Klason lignin and shown to contain significantly less acid-insoluble lignin than WT or CTL tissues (49% and 37% less, respectively; Fig. 3B; Supplementary Table S3 at *JXB* online). Moreover, the RNAi1-1 tissues contained significantly less acid-soluble lignin compared with the WT (21% less on average). Overall, the stems of RNAi1-1 and RNAi1-3 plants had, on average, 43% and 30% less lignin than the respective control plants, both values of which were significant (Fig. 3B; Supplementary Table S3).

Thioacidolysis was employed to quantify the relative amounts of S, G, and H units in the polymeric lignin (Lapierre, 1993; Robinson and Mansfield, 2009), and it was shown that both the RNAi1-1 and 1-3 senesced stems had significantly higher S:G ratios along with significantly higher relative amounts of H units in lignin, compared with the respective control plants grown concurrently (Fig. 3B; Supplementary Table S3 at *JXB* online). The observed S:G ratio increase, which is probably a consequence of altered relative fluxes through the S and G branches of the monolignol biosynthetic pathway, was similar to that observed in *Arabidopsis pal1 pal2* double mutant plants (Rohde *et al.*, 2004). Wang *et al.* (2014) postulate that the higher S:G ratio observed in dicot *pal* mutants, which is paradoxical given that PAL acts upstream of the branch point to S and G biosynthesis, is a consequence of the kinetic properties of enzymes in each biosynthetic branch. Whether the kinetic properties of the corresponding enzymes in *Brachypodium* are similar to those found in *Arabidopsis* remains to be established in future studies.

### *BdPAL* knockdown greatly affects hemicellulose ferulate content, but only modestly affects relative cellulose and hemicellulose amounts

The cell walls of grasses, unlike the cell walls of most dicot species, incorporate FA and *p*CA appendages on hemicelluloses along with *p*CA appendages on lignin. To determine if *BdPAL1* and/or *BdPAL2* are responsible for or influence FA and *p*CA formation, their respective concentrations were assessed. The data (Fig. 3C; Supplementary Table S3 at *JXB* online) show that RNAi1-1 plant cell walls had on average 57% and 16% reductions in the amounts of released FA and *p*CA, respectively, compared with the WT. On the other hand, the differences in FA and *p*CA released from RNAi1-3 stem tissues were not significant, suggesting that the knockdown of *BdPAL1* and/or *BdPAL2* activity in RNAi1-3 plants may not have surpassed a threshold necessary to produce these differences.

Quantitation of component carbohydrate monomers showed that *BdPAL* RNAi1-1 and RNAi1-3 stems contained slightly higher levels of glucose and xylose, the main structural sugars in *Brachypodium* polysaccharides, compared with controls grown concurrently (Table 3). More significantly, *BdPAL* RNAi1-1 and RNAi1-3 stems were both found to have greater amounts of arabinose (33% and 16% more on average, respectively) and galactose (20% and 9% more, respectively) compared with controls. There were no apparent differences in the other minor sugars tested—mannose, rhamnose, and fucose—except for significantly more mannose in RNAi1-3 stems (*P*<0.01; Table 3). Taken together, whereas these data suggest that stem cell walls in *BdPAL* RNAi1 plants had slightly more matrix polysaccharides compared with controls, the differences for the main structural sugars were either insignificant or borderline-significant, suggesting that differences were a consequence of the lower lignin as opposed to an active compensation by the plants for the reduced lignin by increasing hemicellulose biosynthesis. That being said, the increased arabinose levels in both *BdPAL* RNAi lines were highly significantly different compared with the respective controls (*P*=0.0002 and *P*=0.002 for RNAi1-1 and RNAi1-3, respectively). Arabinose is a constituent in the arabinoxylan component that is covalently linked to the FA that is crucial in stabilizing the wall (Ralph *et al.*, 2004*a*; de O Buanafina, 2009; Ralph, 2010). Compensation for the reduced lignin by increasing feruloyl arabinose incorporation in order to increase FA cross-linking is one possible explanation. Feruloylated arabinans are also implicated in rhamnogalacturonan I (RG1), as are galactans, so the increases in both arabinose and galactose may implicate pectin differences; pectins have also recently been shown to influence saccharification (Francocci *et al.*, 2013; Xiao and Anderson, 2013; De Souza *et al.*, 2015).

**Table 3.** Weight percent amounts of sugars released from cell wall polysaccharides, and relative cell wall crystallinity, comparing BdPAL RNAi knocked-down stem tissue with the respective control WT and RNAi1-1 plants were grown together and should be compared with each other, as were CTL and RNAi1-3.

Sample	Glucose	Xylose	Arabinose	Galactose	Mannose	Rhamnose	Fucose	Crystallinity index
WT	42.4±1.0	25.0±0.5	2.99±0.07	0.68±0.03	0.12±0.01	0.074±0.007	0.034±0.002	0.341±0.002
RNAi1-1	44.3±0.8	26.1±.0.3	3.97±0.11	0.82±0.02	0.14±0.01	0.090±0.009	0.038±0.003	0.360±0.011
*P*-value	0.06	0.03	0.0002	0.002	0.18	0.09	0.12	0.04
CTL	43.7±0.9	24.9±.0.7	2.83±0.06	0.70±0.03	0.13±0.004	0.085±0.008	0.033±0.002	0.324±0.002
RNAi1-3	46.4±0.7	26.1±.0.4	3.29±0.09	0.76±0.02	0.14±0.002	0.078±0.021	0.030±0.002	0.340±0.012
*P*-value	0.02	0.1	0.002	0.03	0.007	0.6	0.23	0.09

Values are means ±SD. *P*-values comparing the knock-down line with its control were calculated using the Student’s t-test. *n*=3 independent pools of senesced stem plus leaf sheath tissues (three technical reps for each).

To determine if the *BdPAL* RNAi plants may have compensated for the reduced lignin by producing more cellulose, X-ray diffraction was employed to quantify the cell wall crystallinity. The data in Table 3 showed that the RNAi1-1 knocked-down plant biomass had slightly increased crystallinity compared with the respective controls. This attribute was probably related to the reduced lignin content in the cell wall and not an up-regulation of cellulose biosynthesis. Consistent with this hypothesis, RNA-seq analysis found no significant differences in expression of putative *CELLULOSE SYNTHASE A* (*CESA*; *BdCESA4*, *7*, and *8*) genes in *BdPAL* RNAi1-1 stem tissues compared with CTL (Supplementary Table S4 at *JXB* online) (Handakumbura *et al.*, 2013). Notably, a recent report in *Arabidopsis* showed that plants having a 36% reduction in lignin had no compensatory increase in cellulose; that study did find an increase in matrix polysaccharides (Van Acker *et al.*, 2013).

### Reduced *BdPAL* function results in expression changes of other known and putative monolignol biosynthetic genes

As noted above, the PlaNet gene network prediction tool predicted that *BdPAL1* and *BdPAL2* are co-expressed with a number of other putative monolignol biosynthetic genes. To determine whether these genes had altered expression in *BdPAL* RNAi1-1 stem tissues, as was observed in *Arabidopsis pal* mutants (Rohde *et al.*, 2004), their RNA-seq-derived average transcript levels were compared in *BdPAL* RNAi1-1 stem tissues versus the CTL. Supplementary Table S5 at *JXB* online shows that the average transcript abundance associated with Bradi2g36910 (encodes *BdPMT*), Bradi2g21300 (encodes a putative *C3′H*), and Bradi3g06480 (encodes *CAD*) were significantly higher in *BdPAL* RNAi1-1 stem plus leaf sheath tissues compared with the corresponding average transcript levels in CTL stem plus leaf sheath tissues (*P*<0.05). Using the odds ratio and Fisher’s exact test analysis, it was found that the odds of the putative monolignol biosynthetic genes listed in Supplementary Table S5 being overexpressed in *BdPAL* RNAi1-1 plants were 24-fold higher compared with genes that were not in that set, a difference that was highly significant (Fisher’s exact test *P*=0.0006 when comparing expression of the 10 PlaNet-derived putative monolignol biosynthetic genes, and *P*=6.11×10^–5^ when comparing expression of all 13 genes listed in Supplementary Table S5). One can speculate that this result indicates that both monocot and dicot plants sense the reduced lignin, and/or that monocots sense the reduced cell wall FA cross-linking, and/or the related mechanical or physiological stress (Monshausen and Haswell, 2013), and attempt to increase lignin and/or FA production by up-regulating key biosynthetic gene isoforms. If so, then those isoforms would be good candidates for further study.

### *BdPAL* RNAi plants grew more slowly than the WT

**Fig. 4.**
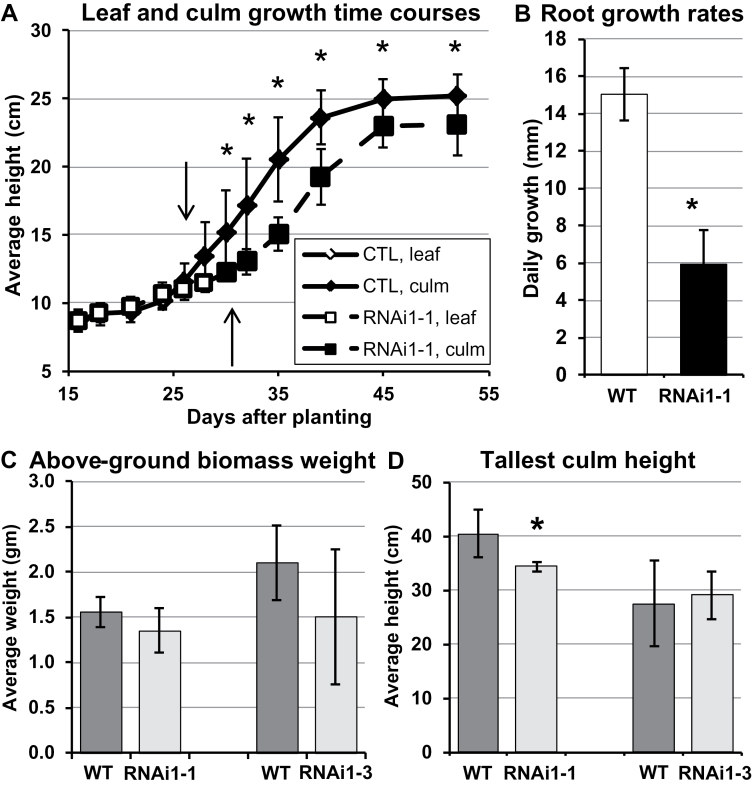
Growth of *BdPAL* RNAi1-1 and 1-3 plants compared with the WT or empty vector control (CTL). (A) Time course measurements of the longest leaf lengths and tallest culm heights of *BdPAL* RNAi1-1 plants. Arrows delineate the time points at which 50% of the spikelets could be seen emerging from the leaf whorl. (B) Average daily root growth of *BdPAL* RNAi1-1 and WT seedlings. *n*=48. (C, D) Average above-ground senesced biomass dry weight (C) and final culm heights (D). Columns grouped together represent plants grown concurrently. Bars denote the SD. Asterisks represent Student’s *t*-test significant differences (*P*<0.03). 5<*n*<25.

To determine if *BdPAL* RNAi1-1 and 1-3 plants were affected in growth and development, lengths of leaves and culm heights were measured over time, revealing a slight but significant delay in growth of plants from both lines compared with the CTL (Fig. 4A; Supplementary Fig. S5A at *JXB* online). The culms of RNAi1-1 and 1-3 plants typically emerged and flowered a few days later than did CTL culms, suggestive of developmental delays. Moreover, RNAi1-1 and 1-3 culms grew to heights that, on average, were slightly shorter than the WT or CTL. RNAi knocked-down plants also accumulated slightly less above-ground biomass. Although the differences were not always statistically significant (Fig. 4B, C), they were significant in some experimental replicates, suggesting that the degree of *PAL* knockdown in these plants may be near the threshold of causing a yield penalty under controlled growth chamber conditions. More significantly, root growth assays revealed that *BdPAL* RNAi1-1 roots grew, on average, 60% more slowly than WT roots (Fig. 4B). While the underlying cause of the reduced root growth remains unclear, less extensive root systems could adversely impact above-ground growth.

Although RNAi1-1 and 1-3 plants looked morphologically similar to the WT, at times they developed lesions on their leaves suggestive of fungal infection; in particular, blackened spots or blackened zones located at the leaf collars (Supplementary Fig. S5B–E at *JXB* online). These lesions were not observed on WT or CTL plants grown intermixed in climate-controlled growth chambers.

### *BdPAL* RNAi1-1 plants have greatly enhanced fungal pathogen susceptibilities

**Fig. 5.**
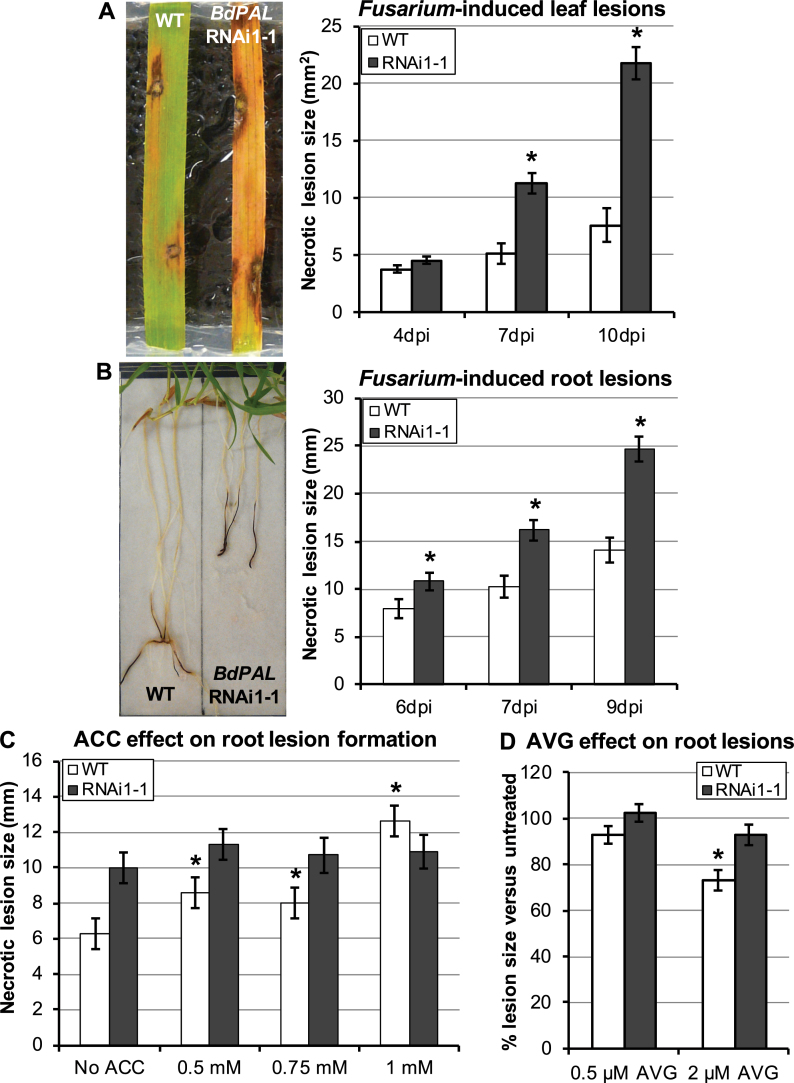
Effect of *BdPAL* silencing on *Fusarium* disease resistance. (A) Comparison of the foliar necrotic lesions that developed on wounded leaves of WT Bd21-3 and *BdPAL* RNAi1-1 following *F. culmorum* infection. The image shows necrotic lesions 7 days post-infection (dpi). *n*=32. (B) Comparison of the root necrotic lesion sizes developed on WT and *BdPAL* RNAi1-1 roots following *F. culmorum* infection. 22<*n*<24. Image shows seedlings 9 dpi. Asterisks in (A) and (B) represent Student’s *t*-test *P*-value <0.001, comparing *BdPAL* RNAi1-1 with the WT for a given time point. (C, D) Comparison of the effect of ACC (C) and AVG (D) treatments on the necrotic lesion sizes developed on WT and *BdPAL* RNAi1-1 roots, 7 d following *F. culmorum* infection. 20<*n*<24. For (C, D), asterisks represent Student’s *t*-test *P*-values <0.001, which relate to the comparison of each treatment with its corresponding untreated control. For all graphs, error bars represent the standard error (SE).

*Brachypodium distachyon* was recently shown to display a compatible interaction with *F. culmorum*, one of the predominant causal agents of *Fusarium* diseases in cereals (Peraldi *et al.*, 2011). To assess the effects that *BdPAL* silencing might have on fungal disease resistance, leaf and root tissues of *BdPAL* RNAi1-1 plants were infected with *F. culmorum* conidial inoculum. When *F. culmorum* conidia were deposited on punctured leaf sites, no significant difference in necrotic lesion formation was observed up to 4 d following infection. However, thereafter, lesions developed more rapidly and became significantly larger on *BdPAL* RNAi1-1 leaves compared with WT Bd21-3 (*P*<0.001; Fig. 5A). Moreover, whereas chlorosis developed around necrotic lesions in both lines, it was strikingly more extensive on *BdPAL* RNAi1-1 leaves (Fig. 5A).

Disease susceptibility of *BdPAL* RNAi1-1 plants was further characterized in the roots. Following infection of root tips with *F. culmorum*, the average length of necrotic lesions that developed on *BdPAL* RNAi1-1 roots was significantly larger compared with WT roots (*P*<0.001 at all time points; Fig. 5B).

The effect of *BdPAL* knockdown on disease resistance was further examined with a different fungal pathogen to test whether the effect on resistance was specific to *F. culmorum* or more general. *Magnaporthe oryzae*, the causal agent of rice blast disease, was the first fungal pathogen for which a compatible interaction was demonstrated with *Brachypodium* (Routeledge *et al.*, 2004). *BdPAL* RNAi1-1 plants sprayed with *M. oryzae* developed a significantly greater number of necrotic lesions per square millimetre compared with the WT (Supplementary Fig. S6 at *JXB* online; *P*<0.001), demonstrating that *BdPAL* knockdown also increases susceptibility to *M. oryzae*.

### *Fusarium* exploits ethylene signalling in *Brachypodium*; however, the increased *BdPAL* RNAi1 fungal susceptibility is probably not due to ethylene

Previous chemical studies showed that *Fusarium graminearum* exploited ethylene signalling to infect barley and wheat, as it does when infecting *Arabidopsis* (Chen *et al.*, 2009). To determine whether exogenous applications of the ethylene precursor ACC also increase the susceptibility of WT *Brachypodium* plants to *Fusarium* infection, roots were treated for 6h with various concentrations of ACC prior to inoculation with *F. culmorum*. The results in Fig. 5C show that 0.5, 0.75, and 1mM ACC treatments of WT roots resulted in *F. culmorum*-induced lesion sizes that were significantly larger than those that formed on WT roots not treated with ACC. The effect of 1mM ACC treatment was dramatic, resulting in a doubling of the average lesion size (*P*<0.001). In contrast to the WT, the susceptibility of the *BdPAL* knocked-down line was not increased by enhancing ethylene biosynthesis through amendment with ACC (Fig. 5C), suggesting that ethylene-associated susceptibility to *F. culmorum* was already saturated in the *BdPAL* RNAi1-1 line or that *BdPAL* knockdown resulted in susceptibility that somehow masked ethylene-associated susceptibility.

To assess further the role played by ethylene signalling with respect to increased susceptibility to *F. culmorum* infection in *BdPAL* RNAi1-1 lines, ethylene biosynthesis inhibition via AVG treatment was tested on WT and *BdPAL* RNAi1-1 seedlings. Two days following *F. culmorum* infection, necrotic lesions on WT roots were relatively smaller than those on WT roots not treated with AVG, the reduced size associated with 2 μM AVG pre-treatment being 27% (*P*<0.001). In contrast, lesion sizes on *BdPAL* RNAi1-1 roots treated with 2 μM AVG were not significantly different from those on untreated roots, being only 7% smaller (Fig. 5D). Although the WT results confirmed that ethylene signalling is a susceptibility factor for *F. culmorum* infection in *Brachypodium*, the absence of a significant effect of AVG upon development of necrotic lesions on *BdPAL* RNAi1-1 roots suggests that susceptibility was largely independent of ethylene signalling.

### *BdPAL* RNAi1-1 plants generally exhibited WT susceptibilities to herbivory

As *BdPAL* RNAi1-1 plants had the strongest phenotypes, plants from that line along with WT accession Bd21-3 plants were used to assess susceptibility to fall armyworm (*Spodoptera frugiperda*) and corn earworm (*Helicoverpa zea*) herbivory. Green leaves and stems removed from plants beginning to form flower heads were caged separately with first instar larvae for 2 d, after which herbivory was quantified. Whereas the amount of fall armyworm leaf feeding damage on *BdPAL* RNAi1-1 plant leaves was significantly lower than that for WT plants (*F*=8.05, *P*=0.0067), there were no significant differences in feeding damage by corn earworms for the two different plant types (Table 4). There also were no significant differences in mortality for either fall armyworms or corn earworms when fed on the leaves of the two different plant types (Table 4). However, there were significant correlations between feeding damage and mortality (i.e. lower feeding damage was associated with higher mortality) for both WT (*F*=11.51, *P*=0.0048) and *BdPAL* RNAi1-1 (*F*=7.00, *P*=0.0125) for fall armyworms. Fall armyworms fed on *BdPAL* RNAi1-1 leaves were significantly larger than those fed on WT leaves, but corn earworms were not. This suggests the *BdPAL* knocked-down leaves may be more nutritious to fall armyworms, allowing caterpillars to obtain required resources from less leaf material. Slansky (1993) found that insects ‘sense’ nutrient levels and adjust their feeding rates accordingly. Moreover, dilution of nutrients with non-nutritive components, such as cellulose, can produce a compensatory feeding response (Lee *et al.*, 2004), a phenomenon shown to occur in the fall armyworm (Wheeler and Slansky 1991). Feeding damage, survivorship, and weights of larvae were also determined in the assays where larvae were caged with stems of the WT and *BdPAL* RNAi1-1 plants. There were no significant differences in mortality, feeding levels, or weights of survivors of fall armyworms or corn earworms when caged with stems from the different plants (Table 4). Thus, there was some indication for fall armyworms that PAL inhibition increased the nutritional content of leaves, but there was no evidence for a significant effect on stems for either of them.

**Table 4.** Effect of BdPAL RNAi1-1 leaves and stems on corn earworms (CEW) and fall armyworms (FAW) at equivalent developmental stages

Herbivory	Line	*n*	Leaf feeding			Mortality (%)	Weight (mg)
CEW	WT	15	32.8±2.0 a			1.5 a	1.22±0.08 a
	RNAi1-1	38	33.4±1.6 a			0.3 a	1.33±0.05 a
FAW	WT	15	67.3±1.9 a			7.2 a	1.38±0.08 a
	RNAi1-1	38	57.5±2.1 b			12.5 a	1.69±0.08 b
			**Stem feeding**				
			**Total**	**Pith**	**Side**		
CEW	WT	15	33.3 a	26.7 a	6.7 a	4.0 a	0.58±0.05 a
	RNAi1-1	31	43.8 a	25.0 a	18.8 a	3.7 a	0.64±0.03 a
FAW	WT	15	33.3 a	33.3 a	0.0 a	0.0 a	0.58±0.03 a
	RNAi1-1	31	59.4 a	56.2 a	9.4 a	0.5 a	0.55±0.03 a

Feeding values are from day 2 and are based on either numbers of 0.25mm^2^ hole equivalents (leaves) or percentage of stems showing feeding damage at the respective locations. Weights are means ±standard errors (SE) in milligrams, and are based on total larval weight per dish. Values of like assays followed by different letters are significantly different at *P*<0.05 by χ^2^ (mortality) or analysis of variance (feeding rating, weights).

### *BdPAL* RNAi plant growth responses to UV light and drought stress were indistinguishable from WT responses

The expression levels of various *PAL* isoforms present in both dicot and monocot plant species have been found to change substantially in response to a variety of abiotic and biotic challenges, including UV irradiation, drought, wounding, and pathogen infection (Camm and Towers, 1973; Edwards *et al.*, 1985; Liang *et al.*, 1989; Dixon and Paiva, 1995; Liu *et al.*, 2010; Moura *et al.*, 2010; Giberti *et al.*, 2012). In *Arabidopsis*, loss-of-function *pal1 pal2* double mutant plants were shown to exhibit substantially increased sensitivity to short-term UV-B treatment and substantially increased tolerance to drought stress (Huang *et al.*, 2010). To determine if *Brachypodium BdPAL* RNAi1-1 plants had similar differences in abiotic stress tolerances, RNAi knocked-down and either WT or CTL plants were subjected to UV-B and drought, using experimental designs similar to those used on *Arabidopsis pal1 pal2* plants (Huang *et al.*, 2010).

To identify a UV-B dose that had an intermediate effect on WT *Brachypodium* plants, WT soil-growing plants were exposed to 2, 4, 6, and 8h of high-intensity UV-B light. Plants exposed for 2h subsequently showed few signs of tissue damage, whereas plants exposed for 8h exhibited severe symptoms within 24–48h, having most leaves turn brown and leaf blades rolling adaxially along the leaves’ axes. As exposure of WT plants for 4h resulted in modest tissue damage, that dosage was chosen to interrogate *BdPAL* RNAi1-1 plants. As seen in the representative images in Supplementary Fig. S7 at *JXB* online, the degree of UV-B-induced damage to *BdPAL* RNAi1-1 plants was indistinguishable from that of the WT. Both *PAL* knocked-down and WT plants developed comparable amounts of tissue browning and leaf curling over the same period of time (Supplementary Fig. S7). Although the leaves of some *BdPAL* RNAi1-1 plants appeared to roll more than those of the WT, the difference was very subtle and might have been instead due to developmental differences between RNAi1-1 and WT plants.

To assess drought stress tolerance, *BdPAL* RNAi1-1, RNAi1-3, and CTL plants were grown intermixed in 4 inch potting soil-containing pots and were well watered until just before culm emergence. The pots were then left in the growth chamber unwatered, and plants were closely monitored and scored for the hallmark signs of drought stress (leaf blade rolling, chlorosis, wilting, and drying). As seen in Supplementary Fig. S8 at *JXB* online, *BdPAL* RNAi1-1 plants looked indistinguishable from empty vector CTL plants, except for being slightly smaller, which was a phenotype not related to the drought stress. Careful examination did identify slightly more leaf rolling in the *BdPAL* RNAi1-1 plants, but, like the UV-induced phenotypes, the differences between RNAi knocked-down and WT plants were subtle and could be attributable to developmental variation. No significant differences were observed in the time it took leaves in *BdPAL* RNAi1-1 and 1-3 plants to start rolling compared with CTLs, nor were there differences in how rapidly the plants progressed through wilting, drying, and death. These indistinguishable phenotypic differences between *BdPAL* knocked-down and CTL plants were in stark contrast to the unambiguous drought-tolerant phenotype observed in *Arabidopsis pal1 pal2* double mutant plants (Huang *et al.*, 2010).

### RNA-seq expression analysis implicates candidate genes involved in the phenylpropanoid pathway and related stress responses

RNA-seq analysis of gene expression in *BdPAL* RNAi1-1 versus WT stem plus leaf sheath tissues uncovered 153 genes whose average expression levels were significantly different, based on an adjusted *P*-value cut-off of 0.05 (Supplementary Table S7 at *JXB* online). Among these 153 genes were several that are predicted to be involved in abiotic and biotic stress responses, phenylpropanoid biosynthesis, cuticular wax biosynthesis, photosystem function, hormone responses, etc. To obtain a clearer picture as to what cellular processes might be affected in *BdPAL* RNAi1-1 plants, a Generally Applicable Gene-set Enrichment (GAGE) analysis (Luo *et al.*, 2009) of the RNA-seq data was run, using MapMan gene sets to delineate which genes may be involved in a given process (Thimm *et al.*, 2004; Luo *et al.*, 2009). The output, which can be found in Supplementary Table S6, suggests that a number of gene sets were misregulated in the *BdPAL* RNAi1-1 stem plus leaf sheath tissues, including those involved in aromatic amino acid, flavonoid, and isoprenoid metabolism, ethylene synthesis and signal transduction, and jasmonate synthesis. If confirmed by functional analyses, increased jasmonate synthesis and signalling could partially explain why *BdPAL* RNAi1-1 plants did not exhibit increased susceptibility to herbivory. Moreover, the GAGE analysis revealed that a number of genes involved in photosystem light reactions were down-regulated in the *BdPAL* knocked-down plants. Reduced photosynthetic capacity could partially explain why *BdPAL* RNAi1-1 plants grew relatively more slowly than the WT.

It should be noted that some of the genes designated by MapMan as being involved in a given process or pathway may be misannotated. Therefore, additional work must be done to check and validate each candidate gene.

## Discussion

A search of the *Brachypodium* genome revealed eight putative *PAL* genes with conserved homologies, suggesting that they encode enzymatically active ammonia lyases that utilize phenylalanine, tyrosine, and/or closely related substrates. Expression and co-expression analyses suggested that two of the *Brachypodium PAL* genes, which were named *BdPAL1* (Bd3g49250) and *BdPAL2* (Bd3g49260), probably both play prominent roles in lignin biosynthesis. Both *BdPAL1* and *BdPAL2* are expressed in lignin-forming stem tissues at substantially higher levels than the other six *BdPAL* genes (Table 2), and both are uniquely co-expressed with other genes known to be involved in lignin biosynthesis (Supplementary Tables S1, S2 at *JXB* online). qRT–PCR analysis revealed the *BdPAL2* gene to be reduced by relatively the same amounts in *BdPAL* RNAi1-1 and 1-3 stem plus leaf sheath tissues, whereas *BdPAL1* gene expression was significantly lower in *BdPAL* RNAi1-1 (Fig. 1). These data suggest that the stronger *BdPAL* RNAi1-1 phenotypes, including reduced cell wall FA and *p*CA and increased digestibility, were a consequence of this greater *BdPAL1* knockdown.

Interestingly, the maize PAL protein GRMZM2G074604, which shares the highest sequence identity with BdPAL1 (Supplementary Fig. S2 at *JXB* online), was shown to utilize both tyrosine and phenylalanine as substrates (Rösler *et al.*, 1997). Like GRMZM2G074604 and other monocot PAL proteins that are bifunctional in regard to substrate specificity, BdPAL1 harbours a histidine residue (His123) in the predicted active site. In contrast, the other BdPAL proteins harbour a phenylalanine residue at this position. All enzymatically tested PAL proteins that have a phenylalanine residue at this site specifically utilize phenylalanine as a substrate (Watts *et al.*, 2006). Therefore, BdPAL1 may be the only bifunctional PAL/TAL protein in *Brachypodium*. The observation that both PAL and TAL activities were reduced in *BdPAL* RNAi1 plant tissues is consistent with BdPAL1 being bifunctional, although additional enzymatic studies must be done to confirm conclusively that BdPAL1 is the sole *Brachypodium* PAL/TAL protein.

Even though the RNAi construct had sufficient coding sequence homologies with all eight *BdPAL* genes conceivably to target reduced expression of any or all of them, the RNA-seq data showed that the expression levels of only *BdPAL1* and *BdPAL2* were reduced in *BdPAL* RNAi1-1 stem plus leaf sheath tissues, whereas the expression level of *BdPAL4* increased (Table 2). More work is required to assess expression levels of these genes in all tissue types and to determine which respond to environmental stimuli and/or in various phenylpropanoid pathway-compromised genetic backgrounds.

Giberti *et al.* (2012) used semi-quantitative RT-PCR to assess expression of putative *PAL* genes in rice suspension cultured cells, finding that the expression of two (*PAL04* Os05G35290 and *PAL07* Os04G43800) was induced by exposure to cell wall hydrolysates derived from the rice blast-causing fungal pathogen *M. oryzae*. It will be instructive to determine which *Brachypodium PAL* isoforms might also be pathogen induced; BdPAL3 (Bd3G49270) and BdPAL8 (Bd5G15830) are the two PAL isoforms sharing the highest sequence identities to rice PAL04 and PAL07, respectively (Supplementary Fig. S1 at *JXB* online). Tonnessen *et al.* (2015) recently reported that a rice mutant line harbouring a deletion in what they designated the *OsPAL4* gene (LOC_Os02g41680) exhibited broad-spectrum susceptibility to bacterial blight, sheath blight, and rice blast. However, it remains unclear whether *OsPAL4* loss of function is the sole underlying cause of the observed mutant phenotypes given that genetic rescue with a WT gene copy was not reported and there were no other *Ospal4* mutants to corroborate the phenotypes.

Although it was found that reducing PAL activity in *Brachypodium* increased the ratio of the S to G lignin units similarly to that observed in *Arabidopsis pal1 pal2* plants (Huang *et al.*, 2010), there were notable differences in how the expression levels of monolignol biosynthetic genes were altered in the *Brachypodium* versus *Arabidopsis pal* loss-of-function mutants. Rohde *et al.* (2004) found that, whereas many monolignol biosynthetic genes were expressed relatively more highly in *Arabidopsis pal* mutants, a *C3′H* isoform and *CCR* isoform were particularly highly expressed, and an *HCT* isoform and *COMT* isoform were much less expressed. In contrast, the present RNA-seq data did not identify any putative monolignol biosynthetic genes as having lower expression in *BdPAL* RNAi1-1 plants versus the CTL (except for *BdPAL1* and *BdPAL2*, which were targeted for reduction), and the putative *C3′H* and CCR isoforms did not stand out as being more highly expressed compared with other putative monolignol biosynthetic genes. It will be instructive to determine if these relative differences between *Arabidopsis* and *Brachypodium* can be tied to differences in how dicots and monocots form cell walls or produce defence compounds.

Compared with the WT, 57% less FA was found to be released from *BdPAL* RNAi1-1 stem cell walls using an alkaline treatment that cleaves the ester bond between FA and arabinoxylan (Fig. 3; Supplementary Table S3 at *JXB* online) as well as between FA and pectins. These data suggest that *BdPAL1* and/or *BdPAL2* are involved in synthesizing FA moieties that become incorporated into cell wall polysaccharides and are involved in cross-linking with other polysaccharides and/or with lignin. This result is consistent with findings by Wakabayashi *et al.* (2012), who showed that PAL activity in rice shoots correlated with cell wall FA and diferulate (DFA) levels. Notably, the rice gene they found to correlate in expression abundance with FA and DFA levels was *OsPAL01* (Os02G41630), which is the rice isoform having the highest sequence identity with *BdPAL1* (Bd3G49250; Supplementary Fig. S1). As such, orthologues of *BdPAL1* in other monocot species appear to be good candidates for contributing to FA production. Consistent with the hypothesized involvement of *BdPAL1* in FA production, significantly reduced FA levels were observed in *BdPAL* RNAi1-1 but not in RNAi1-3 plants; *BdPAL1* was significantly more knocked down in RNAi1-1 versus RNAi1-3 plants, whereas *BdPAL2* was not (Fig. 1B). Further work is required to determine how prominent a role BdPAL1 plays in FA and *p*CA production and whether BdPAL1 is involved in metabolic channelling (the process of passing an intermediary metabolic product directly from one enzyme isoform to another).

The thermochemical and hydrolytic enzyme partial digestion assays identified substantial differences in the amounts of glucose as well as pentose sugars released from *BdPAL* RNAi knockdown plants compared with the WT (Fig. 2). For example, treating ball mill-ground senesced stem biomass with 6.2mM NaOH followed by hydrolytic enzyme digestion resulted in a 93% greater glucose release and a 96% greater pentose sugar release on dry weight yield compared with the WT. Given that the present measurements of total cell wall sugars in *BdPAL* RNAi1-1 extract-free stem biomass identified only 5.8 weight percent more glucose and 7.5 weight percent more pentose sugars compared with the WT (those differences were probably due to the proportional reduction in *BdPAL* RNAi1-1 lignin), it is concluded that the increased sugars yields associated with enzyme digestion were largely due to decreased biomass recalcitrance, with only a modest contribution from the increased amounts of cell wall polysaccharides in *BdPAL* RNAi stems.

Lignocellulosic biomass is recalcitrant to deconstruction due to a combination of the tight packing of cellulose microfibrils and the cross-linking of lignin, hemicelluloses, and pectin polymers, which together hinder hydrolytic enzyme access to and cleavage of polysaccharides (Himmel *et al.*, 2007; DiMartini *et al.*, 2013; De Souza *et al.*, 2015). The present results demonstrated an inverse relationship between Klason lignin amounts and sugar release following saccharification (Figs 2, 3), even with biomass that was not chemically pre-treated. This suggests that the increased digestibility was at least partially due to better enzyme accessibility to the cell wall polysaccharides, which is due in part to the reduced lignin. As noted above, a portion of the increased release of sugars could be attributed to the relatively larger amounts of cell wall polysaccharides in the *BdPAL* RNAi biomass. Another possible factor contributing to the increased digestibility observed with some of the pre-treatments may be a reduction in the amount of FA cross-linking due to the reduction in cell wall FA. It is important to note that alkali pre-treatment readily cleaves FA ester linkages. Therefore, reduced FA cross-linking is probably not responsible for the observed increased digestibility of NaOH-pre-treated *BdPAL* RNAi1-1 biomass. In addition, there may be compositional modifications of deposited hemicellulose polymers and pectin and/or altered branching and related cross-linking profiles contributing to the altered digestibility. In switchgrass and *Miscanthus*, the structure and composition of arabinoxylan and xyloglucan, in addition to lignin amount, were found to affect saccharification yield (DiMartini *et al.*, 2013; De Souza *et al.*, 2015).

The present data show that a >40% reduction in lignin associated with *PAL* knockdown in *Brachypodium* had little effect on abiotic stress tolerance (drought tolerance, UV light tolerance). Similarly, PAL knockdown generally had no significant effect on insect herbivory. In contrast, reduced-lignin *BdPAL* RNAi plants exhibited significantly increased susceptibility to the hemi-biotrophic fungal pathogens *F. culmorum* and *M. oryzae*. Drug studies showed that *F. culmorum* exploited increased ethylene signalling to infect WT Bd21-3 accession *Brachypodium* plants, confirming previous studies (Chen *et al.*, 2009). Although the RNA-seq data indicated that ethylene biosynthesis and signalling were up-regulated in the *BdPAL* RNAi1-1 plants, the drug studies suggest that the increased fungal susceptibility observed in these plants is largely not due to increased ethylene signalling and/or production, as AVG treatment could not rescue necrotic lesions in them as it did in the WT (Fig. 5C). This would suggest that other mechanisms of resistance are compromised by knocking down *PAL* genes. Cell walls are important pre-formed physical barriers that fungal intruders have to overcome for initial penetration as well as for the spread of infection (Walter *et al.*, 2010). Lignin is probably the plant compound most recalcitrant to decomposition by microbes. In addition, FA and *p*CA are known to have strong antifungal activities against *Fusarium* spp. (Bollina *et al.*, 2011; Ponts *et al.*, 2011) and have been associated with resistance to *Fusarium* infection in cereals (Bily *et al.*, 2003; Bollina *et al.*, 2011). Therefore, reduction in lignin, FA, and *p*CA content in *BdPAL* RNAi1-1 cell walls may further facilitate *Fusarium* infection. PAL is also responsible for synthesizing other defence compounds such as flavonoids and chlorogenic acid (Shadle *et al.*, 2003; Treutter, 2006). As such, more work is required to tease apart the underlying susceptibility causes, including studying grass mutants with comparable reductions in lignin that lack secondary metabolite alterations found in *pal* loss-of-function mutants.

It was somewhat surprising that *BdPAL* RNAi plants had UV-B and drought-challenge growth responses that were indistinguishable from those of the WT, given that *Arabidopsis pal1 pal2* plants were found to be substantially more susceptible to UV-B and obviously more resistant to drought (Huang *et al.*, 2010). There could be a number of reasons for these discrepancies. (i) The *Arabidopsis pal1 pal2* plants may have had relatively larger reductions in phenylpropanoid pathway-derived compounds that protect against photo-oxidative damage. (ii) Even though reductions in PAL activity in *Arabidopsis pal1 pal2* and *BdPAL* RNAi1-1 tissues were equivalent [67% reduction in *pal1 pal2* stem tissues as determined by Huang *et al.* (2010), versus 65% reduction in *BdPAL* RNAi1-1 stem tissues], the lignin reductions in the *Arabidopsis pal1 pal2* plants were relatively higher [65% reduction as determined by Rohde *et al.* (2004), compared with the 43% reduction in the *BdPAL* RNAi1-1 plants]. As such, the *Arabidopsis pal1 pal2* lignin reduction may have surpassed a threshold that induced other drought protection mechanisms such as cuticular wax accumulation; (iii) As *Brachypodium* harbours twice as many PAL isoforms as *Arabidopsis*, one or more of those isoforms may specifically function in abiotic stress responses. (iv) Stress-related coping mechanisms may have evolved differently between dicots (*Arabidopsis*) and monocots (*Brachypodium*). It would be interesting to determine if clues can be found to explain these differences by performing comparative transcriptomic and metabolomic analyses.

To the authors’ knowledge, this is the first report of using *B. distachyon* for an insect pest herbivory study. Notably, the fall armyworm and corn earworm feeding rates, percentage mortality, and weight gain reported here are comparable with those observed when this assay was performed with switchgrass, maize, and sorghum tissues under the same conditions (Dowd *et al.*, 2007, 2013; Dowd and Johnson, 2009). Therefore, it seems likely that caterpillar feeding studies with *Brachypodium* could provide important insights into the molecular mechanisms of herbivory resistance and serve as a predictive guide for how comparable genetic changes might behave in economically important grasses. Moreover, the data show that substantially reducing lignin had a minimal effect on herbivory resistance, which is consistent with findings in sorghum, where low-lignin near-isogenic lines generally did not have increased susceptibility to corn earworms or fall armyworms compared with wild-type plants, although pith tissue of these *brown midrib* (*bmr*) 6 and 12 mutants was generally more resistant (Dowd and Sattler, 2015).

Prior studies have investigated the effects of altered PAL expression on different insect and plant combinations. Tobacco plants with reduced PAL expression had reduced levels of systemic acquired resistance, and increased resistance to the tobacco budworm (*Heliothis virescens*), whereas the opposite was true for plants with elevated levels of PAL (Felton *et al.*, 1999). Conversely, silencing of a PAL from wheat resulted in increased susceptibility to Russian wheat aphids (*Diuraphis noxia*; Van Eck *et al.*, 2010). It is not unexpected that up- or down-regulating PAL would produce varying results for different plant and insect species combinations, as PAL is important in the biosynthesis of a variety of defensive compounds that vary in identity and levels in different plant species (Iriti and Faoro, 2009), although effects would probably be more different between dicots and monocots. In addition, many other plant defensive proteins (Dowd *et al.*, 2008) and secondary metabolites (Johnson *et al.*, 2008) play roles in insect resistance and vary from plant to plant.

## Conclusion

These data provide important insights into how knocking down *PAL* in *Brachypodium* and perhaps other grass species affects cell wall formation as well as plant responses to biotic and abiotic challenges. Surprisingly, *Brachypodium PAL* knocked-down plants responded differently to drought and UV light stresses compared with *Arabidopsis PAL* loss-of-function mutants, suggesting that not all phenylpropanoid-related findings in dicots may be translatable to graminaceous species. The RNA-seq data analyses have also uncovered a number of genes that appear to be altered in expression to cope with reduced *PAL* function and/or related cell wall compositional changes. Reduced PAL function had a positive impact on carbohydrate availability but a negative impact on disease resistance. These data could inform gene isoform manipulation choices in economically important grasses, with the goal of improving or maintaining plant fitness while reducing lignin for increased biomass digestibility.

## Supplementary data

Supplementary data are available at *JXB* online.

Supplementary Materials and methods

Figure S1. Neighbor–Joining relatedness tree of known and predicted PAL proteins.

Figure S2. Amino acid sequence alignment of known and predicted PAL proteins.

Figure S3. Alignment of *BdPAL* coding sequences to the RNAi sequence.

Figure S4. PAL and TAL enzymatic activities in *BdPAL* RNAi1-1 roots versus the WT.

Figure S5. Growth time courses and phenotypes of *BdPAL* RNAi plants compared with CTL.

Figure S6. Effect of *BdPAL* silencing on *Magnaporthe oryzae* disease resistance.

Figure S7. WT and *BdPAL* RNAi1-1 plant growth responses to high intensity UV-B light.

Figure S8. CTL and *BdPAL* RNAi1-1 plant growth responses to drought.

Table S1. Genes predicted by PlaNet to be co-expressed with Bradi3g49250 (*BdPAL1*).

Table S2. Genes predicted by PlaNet to be co-expressed with Bradi3g49260 (*BdPAL2*).

Table S3. Lignin compositional analyses of WT and *BdPAL* RNAi senesced stems.

Table S4. Comparison of RNA-seq-derived average transcript levels of putative *CESA* genes in *BdPAL* RNAi stems versus CTL.

Table S5. Comparison of RNA-seq-derived average transcript levels of putative monolignol biosynthetic genes in *BdPAL* RNAi stems versus CTL.

Table S6. GAGE enrichment analysis of MapMan gene sets, comparing RNA-seq-quantified gene transcript levels in *BdPAL* RNAi1-1 stems versus CTL.

Table S7. RNA-seq analysis of transcript levels in *BdPAL* RNAi1-1 stems versus CTL.

Supplementary Data
