# Supplementary material for: Effects of PHENYLALANINE AMMONIA LYASE (PAL) knockdown on cell wall composition, biomass digestibility, and biotic and abiotic stress responses in Brachypodium
Source: J Exp Bot. 2015 Jun 19;66(14):4317–35. doi: 10.1093/jxb/erv269 (PMC4493789; doi:10.1093/jxb/erv269)
Supplement: Supplementary Data [file supp_66_14_4317__index.html]

Effects of PHENYLALANINE AMMONIA LYASE (PAL) knockdown on cell wall composition, biomass digestibility, and biotic and abiotic stress responses in Brachypodium — Supplementary Data 

# Effects of *PHENYLALANINE AMMONIA LYASE* (*PAL*) knockdown on cell wall composition, biomass digestibility, and biotic and abiotic stress responses in *Brachypodium*

## Supplementary Data

Data files

- Supplementary Data - Supplementary Data
- Supplementary Data - Supplementary Data
- Supplementary Data - Supplementary Data
