# Supplementary material for: Effects of PHENYLALANINE AMMONIA LYASE (PAL) knockdown on cell wall composition, biomass digestibility, and biotic and abiotic stress responses in Brachypodium
Source: J Exp Bot. 2015 Jun 19;66(14):4317–35. doi: 10.1093/jxb/erv269 (PMC4493789; doi:10.1093/jxb/erv269)
Supplement: Supplementary Data [file supp_erv269_Supplementary_Data_Cass_et_al.pdf]

SUPPLEMENTARY DATA

Effects of *PHENYLALANINE AMMONIA LYASE* (*PAL*) knockdown on cell wall composition, biomass digestibility, and biotic and abiotic stress responses in Brachypodium

Cynthia L. Cass[1,2], Antoine Peraldi[3], Patrick F. Dowd[4], Yaseen Mottiar[2,5], Nicholas Santoro[6], Steven D. Karlen[2], Yury V. Bukhman[2], Cliff E. Foster[6], Nick Thrower[6], Laura C. Bruno[3], Oleg Moskvin[2], Eric T. Johnson[4], Megan E. Willhoit[1,2], Megha Phutane[1,2], John Ralph[2,7], Shawn D. Mansfield[2,5], Paul Nicholson[3], and John C. Sedbrook[1,2*]

[1]School of Biological Sciences, Illinois State University, Normal, IL 61790, USA
[2]US Department of Energy Great Lakes Bioenergy Research Center, Madison, WI, 53706, USA
[3]Department of Crop Genetics, John Innes Centre, Norwich Research Park, Norwich, NR4 7UH, UK
[4]USDA, Agricultural Research Service, National Center for Agricultural Utilization Research, Crop Bioprotection Research Unit, Peoria, IL 61604, USA
[5]Department of Wood Science, University of British Columbia, Vancouver, Canada
[6]US Department of Energy Great Lakes Bioenergy Research Center, East Lansing, MI 48824, USA
[7]Department of Biochemistry, Wisconsin Energy Institute, University of Wisconsin, Madison, WI, 53706, USA

*Address for correspondence: John Sedbrook, School of Biological Sciences, Illinois State University, Normal, IL 61790, phone 309-438-3374, fax 309-438-3722, email jcsedbr@ilstu.edu

SUPPLEMENTARY MATERIALS AND METHODS

Plant Transformation

A *BdPAL1* RNAi construct targeting the Brachypodium Bd3g49260 gene transcript was made by amplifying a 619 nucleotide fragment from a Brachypodium developing spike EST library clone 5453_B03_D05 ZE5_029 (Vogel et al., 2006) using forward 5′-GGGGGGTACCGGATCCGCATCAACACCCTGCT-3′ and reverse 5′-CAATACTAGTGGCGCGCCAAGCGAATGACTTCGATCT-3′ primers. The amplified fragment was cloned in both orientations flanking the Cre intron in pStarling (BamHI-AscI flanking the sense leg; KpnI-SpeI, the antisense leg). The expression cassette consisting of the maize Ubiquitin promoter plus intron, RNAi legs plus Cre intron, and tml terminator was moved into the pWBVec8 RNAi binary vector as a NotI fragment.

Transgenic Brachypodium plants harboring the *BdPAL* RNAi construct were regenerated from *Agrobacterium tumefaciens*-mediated transformed Bd21-3 embryonic callus tissue as described in Vogel and Hill (2008). All solutions were either autoclaved or filtered through a 0.22 µm filter and stored in single use aliquots. After removing the lemma from green Bd21-3 seeds approximately 54 days after planting in soil, seeds were surface sterilized with 10% (v/v) bleach/0.01% (w/v) SDS for 6 min, washed with sterile water five times, then 0.5-1.5 mm embryos were dissected out in a laminar flow hood, placed on callus initiation media (For 1 L: 4.43 g LS salts/30 g sucrose (Sigma, plant tissue culture tested)/2.5 g Phytagel/1 mL 0.6 mg/mL anhydrous $CuSO_4$, pH 5.8; 0.5 mL of 5 mg/mL 2,4-dichlorophenoxyacetic acid (2,4-D) in 95% (v/v) ethanol added after autoclaving), sealed with Parafilm, wrapped in foil, and placed in a 28°C incubator. Callus pieces were transferred once a week for 3-5 weeks until yellow, friable callus formed.

One to two days prior to callus transformation, Agrobacterium strain AGL-1 harbouring the *BdPAL* RNAi construct was grown on solid MG/L media (For 1 L: 5 g tryptone/2.5 g yeast extract/ 5 g NaCl/5 g mannitol/0.1 g $MgSO_4$/0.25 g K_2HPO_4/1.2 g glutamic acid/15 g Bactoagar/pH 7.2) supplemented with 100 mg/L carbenicillin and 75 mg/L spectinomycin at 28°C in the dark. The AGL-1 was scraped off the plate into 10 mL liquid callus initiation media (above, without the agar and $CuSO_4$) and shaken at 250 rpm at 28°C in the presence of 200 µM acetosyringone in DMSO. After 3 hours, the AGL-1 suspension was diluted to OD_{600}=0.6, supplemented with 10% (w/v) Pluronic F68 (Sigma) at 10 µL per mL bacterial suspension, and

poured over 50-100 approximately 0.5 cm friable callus pieces. Callus pieces were incubated in the Agrobacterium suspension 10 minutes, rocking at least once. After removing as much of the AGL-1 suspension as possible by pipetting, the callus pieces were transferred to sequential sterile Whatman filter papers placed in Petri dishes until no more draining occurred. Plates of callus pieces were sealed with Parafilm, wrapped in foil, and placed in a 22°C incubator.

After three days, the callus pieces were transferred to callus initiation media plates (above) supplemented with 150 mg/L Timentin and 40 U/mL Hygromycin B (Phytotechnology Laboratories), incubated at 28°C, sealed with Parafilm and wrapped in foil. Callus was transferred once a week for 3-4 weeks, discarding necrotic tissue each time. Care was taken to ensure callus pieces did not touch each other from this point onward to ensure that independent event lines arose from separate callus pieces. Healthy white and/or yellow friable callus pieces were then cultured on regeneration media (For 1 L: 4.43 g LS salts/30 g maltose/2.5 g Phytagel pH 5.8 supplemented with 9.3 μM kinetin, 150 mg/L Timentin, and 40 U/mL hygromycin B at 28°C on plates sealed with Parafilm, in a 16:8 hr light:dark cycle, transferring 1X week until green shoots appeared (typically 2-6 weeks). Shoots with 3-4 leaves were transferred to MS media (For 1 L: 4.42 g MS salts plus vitamins/30 g sucrose (above)/2 g Phytagel pH 5.7) supplemented with 150 mg/L Timentin in Magenta boxes at 28°C, 16:8 hr light:dark cycle, until root development occurred (2-4 weeks), and then transferred to soil.

Quantitative RT-PCR (qRT-PCR)

Total RNA was extracted from culm internode (culm plus leaf sheath), culm leaf, or floral tissue from plants grown 35-45 days in soil or roots from plate-grown seedlings by grinding tissue in liquid nitrogen, then purified using a Plant RNeasy RNA extraction kit following manufacturer's directions (Qiagen). 0.5 μg DNaseI-treated (Fermentas) RNA was reverse transcribed using oligo(dT)$_{18}$ and M-MLV (Promega) in a 20 uL reaction volume. The following primer pairs, expected PCR product size, and primer efficiencies were used for qRT-PCR assessment of Bradi3g49250 and Bradi3g49260 transcript levels using a DyNAmo HS SYBR Green qRT-PCR kit (Thermo Scientific): for Bradi3g49250, forward 5′-TGTACCTCACCGGCGAGAAGCTC-3′, reverse 5′-GACCGATGGATACGGGCCAATGGAC-3′, 174 bp, 1.98 ± 0.01; for Bradi3g49260, forward 5′-CTGGCGCCGAACCGGATCAAG-3′, reverse 5′-CTTCAGCTCGATCAGCACAGTGG-3′, 224 bp, 1.94 ± 0.02; and for the reference gene

Bd4g00660 (BdUBC18), forward 5′-ATCCCATGGAGGCACCTCAGGTC-3′, reverse 5′-CGGTCATTGTCTTGCGGACGTTG-3′, 196 bp, 2.01 ± 0.10. First strand cDNA samples were diluted to 2 ng/μL in a 20 μL reaction volume. PCR reactions were analyzed in triplicate by an Applied Biosystems 7300 Real Time PCR system. Thermal cycling conditions: 94 °C for 15 min; 40 cycles of 94 °C for 15 s, 56 °C for 30 s, 72 °C for 30 s; followed by a dissociation stage 95 °C for 15 s, 60 °C for 30 s, 95 °C for 15 s. The ratio gene expression of target genes were determined by normalizing to wild-type samples using an efficiency calibrated model: Ratio = $[(E_{target})^{\Delta Ct\ target}]/[(E_{reference})^{\Delta Ct\ reference}]$ where $\Delta Ct_{target} = Ct_{control} - Ct_{treatment}$ and $\Delta Ct_{reference} = Ct_{control} - Ct_{treatment}$ (Pfaffl, 2001; Equation 1 in Yuan *et al.*, 2006); *BdUBC18* (Bradi4g00660) was used as the reference gene. Primer efficiency (E), defined as $2^{-(1/slope)}$, was calculated from log_2 cDNA concentration vs. Ct value plot slopes of serially-diluted (1:5 dilution) cDNA samples across treatment groups (Yuan *et al.*, 2006) for both target and reference genes.

Growth of Plants

Transgenic seeds were germinated in the presence of Hygromycin B (40 U/mL), plants were grown in a 50:50 mix of SunGro Rediearth and MetroMix 510 soil in 4 inch pots at a density of 5 plants per pot (unless otherwise indicated) in growth chambers (20 h light:4 h dark photoperiod, 22 °C, 50% humidity), and allowed to senesce and dry completely before harvesting. Control plants for all experiments were either non-transformed wild-type Bd21-3 (WT) or empty pWBvec8 vector control (CTL) plants grown alongside the transgenics under identical conditions. For caterpillar herbivory treatments, T_4 seeds from homozygous T3 *BdPAL* RNAi1-1 parents were germinated on non-selective media before planting in soil. Pre-flowering plants were transferred to a plant growth facility in Peoria, IL. In Peoria, plants were held in growth chambers at 25 ± 1 °C., 50 ± 10% relative humidity, and a 14:10 light dark photoperiod, as described previously (Dowd and Lagrimini, 1997). Plants for UV light sensitivity were planted at a density of one plant per 4" pot and exposed 21 days after planting to UV light for 4 h, approximately 5" from the light source. Plants were rotated every hour. Plants for the drought tolerance assay were grown at a density of four plants per 4" pot with two each of CTL and *BdPAL1* RNAi plants on the diagonal in each pot. To ensure equal amounts of soil mix went into each pot, dry soil was weighed and mixed with water for each pot before planting. Soil moisture was measured using a FieldScout TDR100 soil moisture meter (Spectrum Technologies, Inc.).

RNA-seq and gene set enrichment analyses

Aliquots of the RNA prepared for qRT-PCR were used for RNA-seq analysis. Poly(A) RNA and libraries were quality-checked using a 2100 Bioanalyzer (Agilent Technologies) before 2x100bp sequencing with an Illumina HiSeq2000 instrument. The gene expression data was computed using the CLC Genomics Workbench version 6.5. Reads were trimmed and filtered on quality with the Trim Sequences algorithm (Limit: 0.05, Maximum ambiguities: 2). RPKM was generated by aligning ESTs to Gene annotations using the RNA-Seq Analysis algorithm for annotated sequence. (Parameters: Similarity 0.8; Length fraction 0.9). Genome sequence and annotations were downloaded from NCBI (http://www.ncbi.nlm.nih.gov/bioproject/PRJNA74771). Genome sequence, annotation, and expression data were stored in an Oracle relational database and displayed using a custom web application.

Gene set enrichment analysis was done using the GAGE package from Bioconductor (R Core Team, 2014; Gentleman et al., 2004; Luo et al., 2009). Unique count data were normalized by dividing by size factors computed using the 'estimateSizeFactorsForMatrix' function from the DESeq2 package (Anders and Huber, 2010) and taking a log transform as recommended in the gage vignette. Classification of *B. distachyon* genes into sets was obtained from the MapMan resource (Thimm et al., 2004). Enrichment analysis was done separately for each of the top 4 levels of the MapMan classification. Both directional and non-directional tests were performed. If a gene set was detected by both tests, it was reported as "up" or "down" according to the directional test. The sets detected by non-directional test only were reported as "bidirectional".

A separate enrichment analysis was done on a small gene set of known lignin biosynthesis genes defined in house. Enrichment for genes that pass unadjusted DESeq *p*-value threshold of 0.05 and fold change of 1.3 was determined using odds ratio and Fisher exact test, using R function 'fisher.test'.

Pretreatment, saccharification, and sugar quantification (Digestibility assay)

Senesced, ground culm plus leaf sheath biomass from *BdPAL* RNAi, WT, or empty vector control plants was subjected first to a suite of pretreatments, and then to partial hydrolytic enzyme saccharification, followed by colorimetric glucose and pentose quantification (Santoro et al., 2010). Briefly, each biomass sample was automatically ground, weighed, and dispensed in triplicate by a

custom-designed robot known as iWALL (Santoro et al., 2010, Labman Automation Ltd, UK). 750 μL of the various pretreatment solutions were added and incubated at 90°C for 3 hr (except for the "grinding alone" samples, which were incubated in 25°C water for 3 hr), then cooled and neutralized. For hydrolytic enzyme digestion, 50 μL of 30 mM citrate buffer (pH 5.0) containing 0.25 μL Accellerase 1000 (Genencor) in a final volume of 0.8 mL was incubated at 50°C for 20 hours with end-over-end rotation. Solids were removed by centrifugation, then the supernatants assayed for glucose content using the glucose oxidase/peroxidase (GOPOD) method (K-GLUC, Megazyme) with assay volumes reduced to 4 μL of the digestion reaction supernatant and 64 μL of the GOPOD reagent in 384-well microtiter plate. Total pentose was determined as described by Deschatelets and Yu (1986) with assay volumes of 12 μL sample and 56 μL *p*-bromoaniline in thiourea in 384-well microtiter plates. The samples were incubated at room temperature for 70 min, then the absorbance taken at 520 nm. Subsequently, the samples were heated to 100°C for 10 min, incubated at room temperature for 70 min, and the absorbance taken again at 520 nm.

Determination of Free Glucose, Sucrose, and Starch

Stem biomass samples were ground and dispensed as for digestibility samples. For free glucose determination, chemical pretreatment was replaced with distilled water. Samples were treated as above for glucose content determination with a digestion cocktail without Accellerase 1000, and assayed for glucose content using the GOPOD method described above. For sucrose determination, samples were pretreated with 6.25 mM NaOH, heated and cooled as above, then 47.3 μL of an invertase cocktail was added (4 μL Invertase (1U/μL, Sigma-Aldrich)/30 mM citrate buffer (pH 4.5)/0.01% sodium azide) and processed as above. Glucose determination was by the GOPOD method (above) at 25°C. Sucrose results reported are the amount of glucose quantified multiplied by two. For starch determination, samples were treated with 150 μL of 2M KOH, incubated as above, and neutralized with 600 μL 1.2M sodium acetate buffer (pH 3.8). To initiate starch hydrolysis, 60 μL of a solution containing 5 μL amyloglucosidase (K-TSTA, Megazyme, Ireland), 5 μL α-amylase (K-TSTA, Megazyme, Ireland), 50 μL distilled water, and 10 μL 0.01% sodium azide was added, and the samples treated as above using the GOPOD method. Every microplate contained standard curve samples that were used to determine the amount of glucose or pentose in experimental samples. Each biomass sample was assayed in triplicate with each of the triplicate samples quantified in quadruplicate.

SUPPLEMENTARY REFERENCES

Anders S, Huber W. 2010. Differential expression analysis for sequence count data. *Genome Biology* **11**, R106.

Deschatelets L, Yu, E 1986. A simple pentose assay for biomass conversion studies. *Applied Microbiology and Biotechnology* **24**, 379-385.

Dowd PF, Lagrimini LM. 1997. Examination of different tobacco types (Nicotiana spp.) under- and overexpressing tobacco anionic peroxidase for their leaf resistance to Helicoverpa zea. *Journal of Chemical Ecology* **23**, 2357-2370.

Gentleman RC, Carey VJ, Bates DM, Bolstad B, Dettling M, Dudoit S, Ellis B, Gautier L, Ge Y, Gentry J, et al. 2004. Bioconductor: open software development for computational biology and bioinformatics. *Genome Biology* **5**, R80.

Handakumbura PP, Matos DA, Osmont KS, Harrington MJ, Heo K, Kafle K, Kim SH, Baskin TI, Hazen SP. 2013. Perturbation of *Brachypodium distachyon CELLULOSE SYNTHASE A4* or *7* results in abnormal cell walls. *BMC Plant Biology* **13**, 131.

Hsieh LS, Hsieh YL, Yeh CS, Cheng CY, Yang CC, Lee PD. 2011. Molecular characterization of a phenylalanine ammonia-lyase gene (BoPAL1) from *Bambusa oldhamii*. *Molecular Biology Reports* **38**, 283-290.

Hsieh LS, Ma GJ, Yang CC, Lee PD. 2010. Cloning, expression, site-directed mutagenesis and immunolocalization of phenylalanine ammonia-lyase in *Bambusa oldhamii*. *Phytochemistry* **71**, 1999-2009.

Pfaffl MW. 2001. A new mathematical model for relative quantification in real-time RT-PCR. *Nuc. Acids Res.* **29,** 2004-2007.

R Core Team. 2014. R: A language and environment for statistical computing. Vienna, Austria: R Foundation for Statistical Computing.

Santoro N, Cantu SL, Tornqvist C-E, Falbel TG, Bolivar JL, Patterson SE, Pauly M, Walton JD. 2010. A high-throughput platform for screening milligram quantities of plant biomass for lignocellulose digestibility. *Bioenergy Research* **3**, 93-102.

Vogel J and Hill T. 2008. High-efficiency *Agrobacterium*-mediated transformation of *Brachypodium distachyon* inbred line Bd21-3. *Plant Cell Rep* **27**, 471-478.

Vogel J, Gu Y, Twigg P, Lazo G, Laudencia-Chingcuanco D, Hayden D, Donze T, Vivian L, Stamova B, Coleman-Derr D. 2006. EST sequencing and phylogenetic analysis of the model grass *Brachypodium distachyon*. *Theoretical and Applied Genetics* **113**, 186-195.

Yuan JS, Reed A, Chen F and Stewart CN. 2006. Statistical analysis of real-time PCR data. *BMC Bioinformatics* **7**, 85-97.

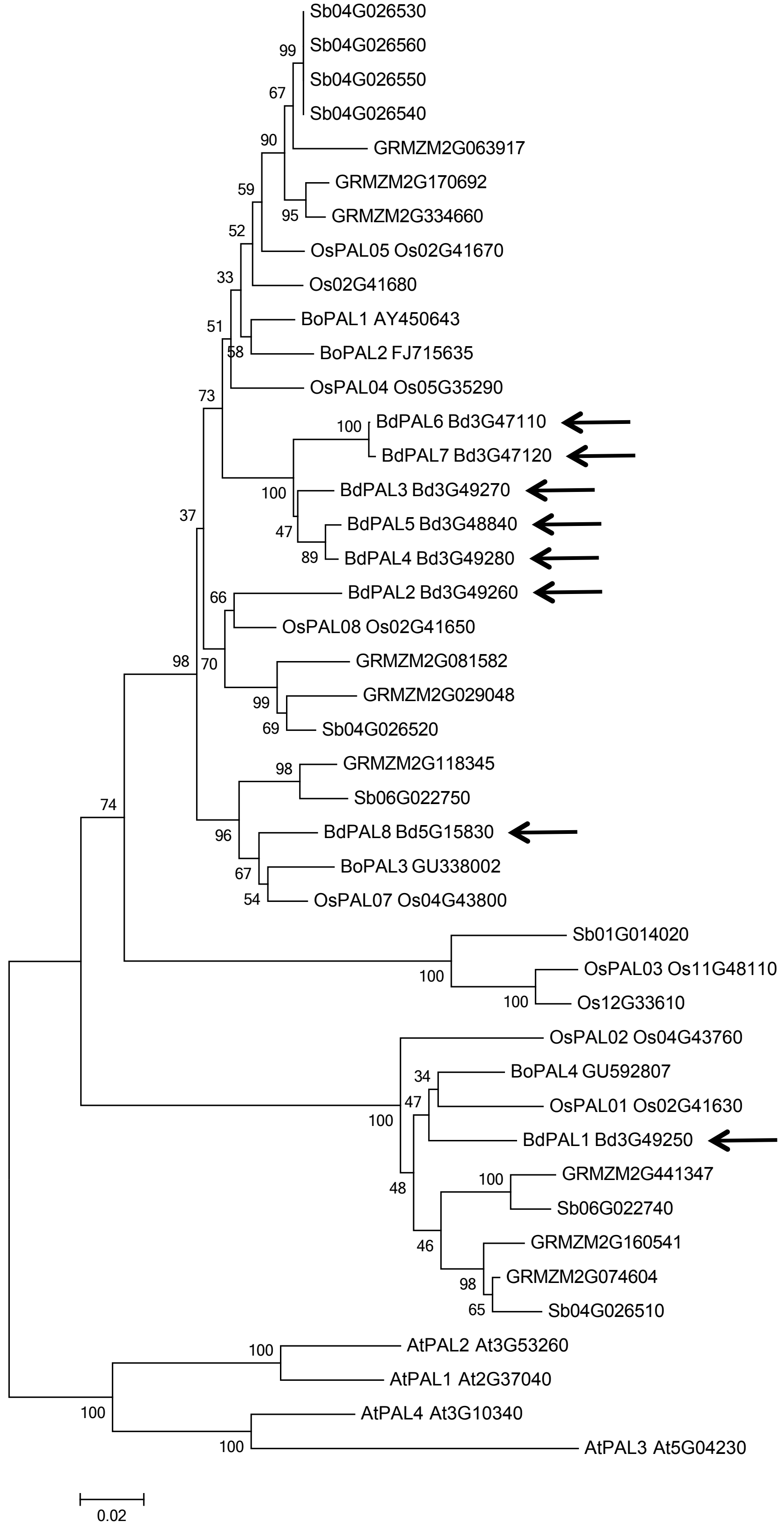

Figure S1. Neighbour-joining relatedness tree of known and predicted PAL proteins. Bd = *Brachypodium distachyon* (marked by arrows); At = *Arabidopsis thaliana* (Huang et al., 2010); GRMZM = *Zea mays*; Sb = *Sorghum* bicolor; Os = *Oryza sativa* (Giberti et al., 2012); Bo = *Bambusa oldhamii* (Hsieh et al., 2010, 2011). Note: Tonnessen et al. (2015) designated the following names for the *Oryza sativa* genes: OsPAL1 LOC_Os02g41630; OsPAL2 LOC_Os02g41650; OsPAL3 LOC_Os02g41670; OsPAL4 LOC_Os02g41680; OsPAL5 LOC_Os04g43760; OsPAL6 LOC_Os04g43800; OsPAL7 LOC_Os05g35290; OsPAL8 LOC_Os11g48110; OsPAL9 LOC_Os12g33610

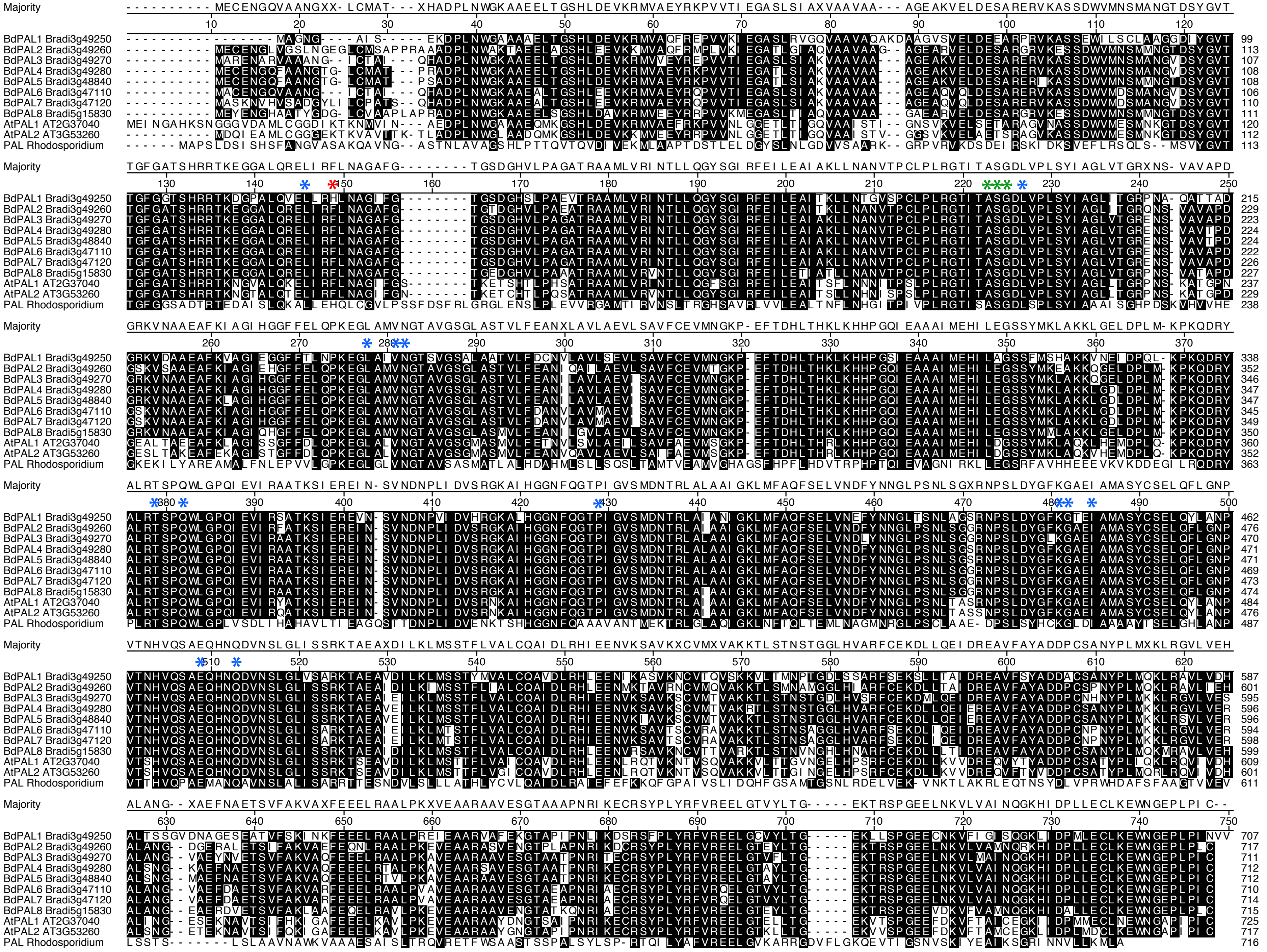

Figure S2. Amino acid sequence alignment of predicted Brachypodium BdPAL proteins, Arabidopsis AtPAL1 and AtPAL2, and the crystal structure-determined *Rhodosporidium toruloides* PAL. Asterisks denote residues highly conserved in the active sites of PAL proteins from diverse species (Calabrese et al., 2004), including the A-S-G amino acid triad that forms the prosthetic group 3,5-dihydro-5-methylidene-4H-imidazol-4-one (MIO; green asterisks). The red asterisk at consensus position 149 denotes an amino acid key in determining substrate specificity (F is selective for phenylalanine, whereas H allows tyrosine as a substrate; Watts et al., 2006). Boxed residues match the consensus.

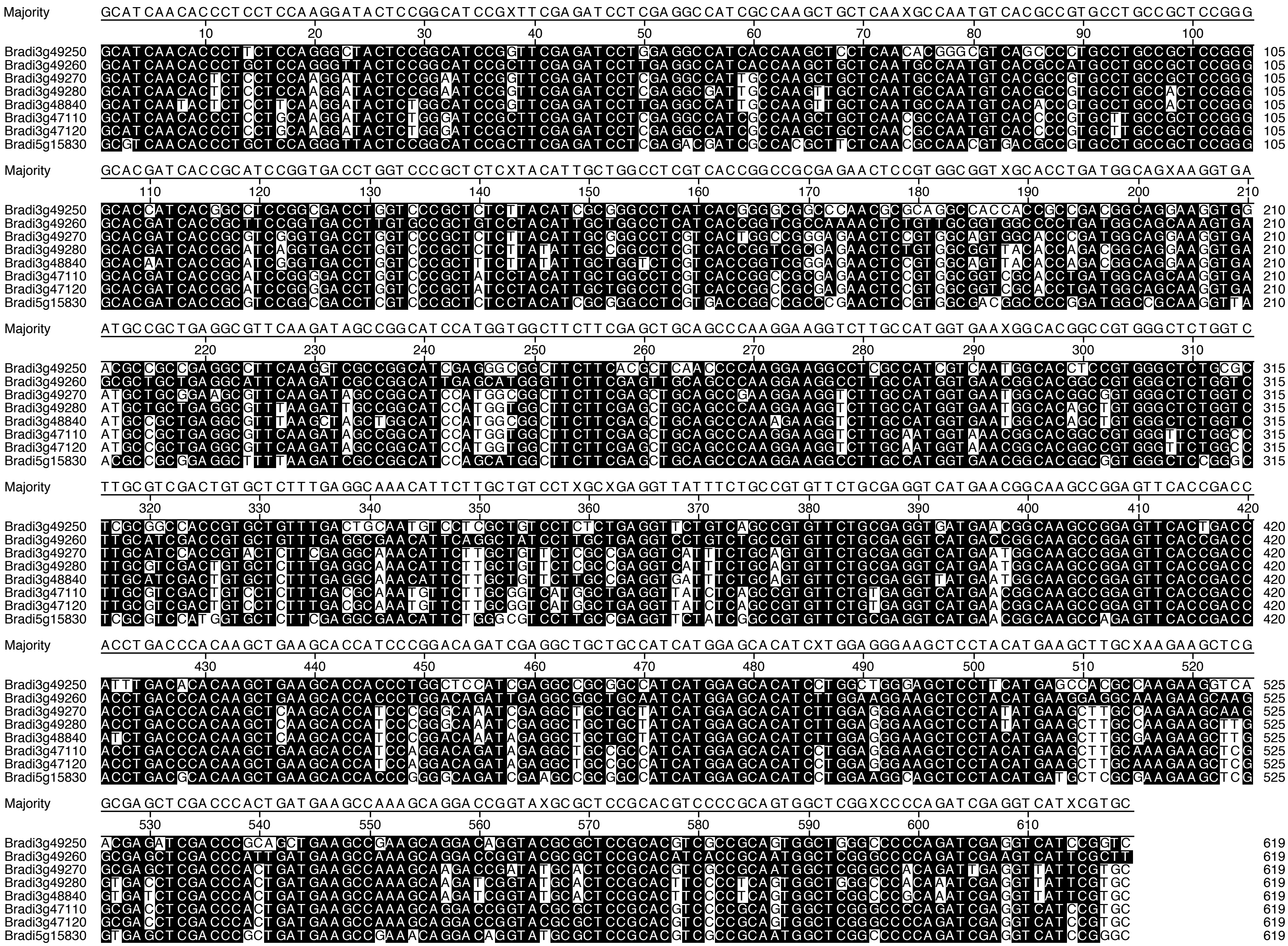

Figure S3. Alignment of the Bradi3g49260 coding sequence comprising the *BdPAL* RNAi construct with the corresponding *BdPAL* genes' coding sequences. Black-boxed nucleotides are identical to the corresponding Bradi3g49260 nucleotides. The Bradi3g49260 sequences are from nucleotides 491 through 1,109 of the 2,154-long Bradi3g49260 (*BdPAL2*) open reading frame.

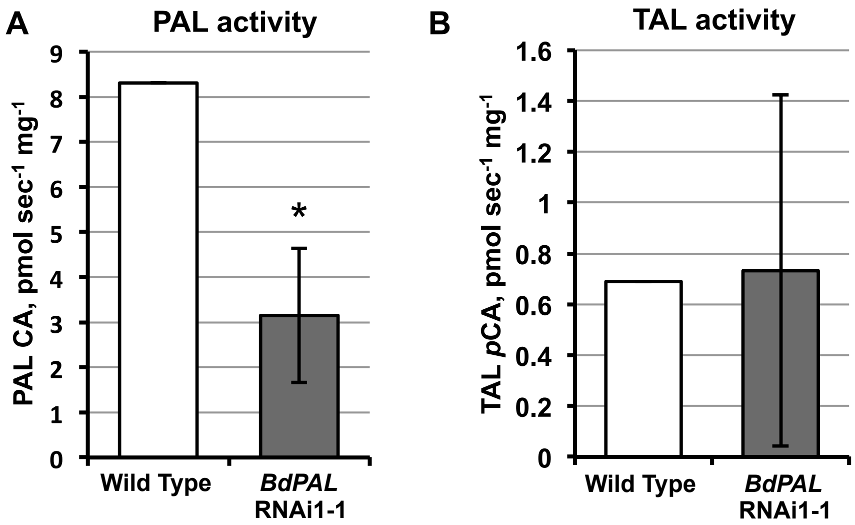

Figure S4. Amounts of PAL activity (**A**) and TAL activity (**B**) in the distal 4 cm of seedlings' roots grown on growth media-containing agar. Bars represent standard deviations (SD). Asterisk represents Student's t-test *p*-value < 0.02 comparing *BdPAL* RNAi1-1 to WT. n = 1 biological rep, 2 technical reps.

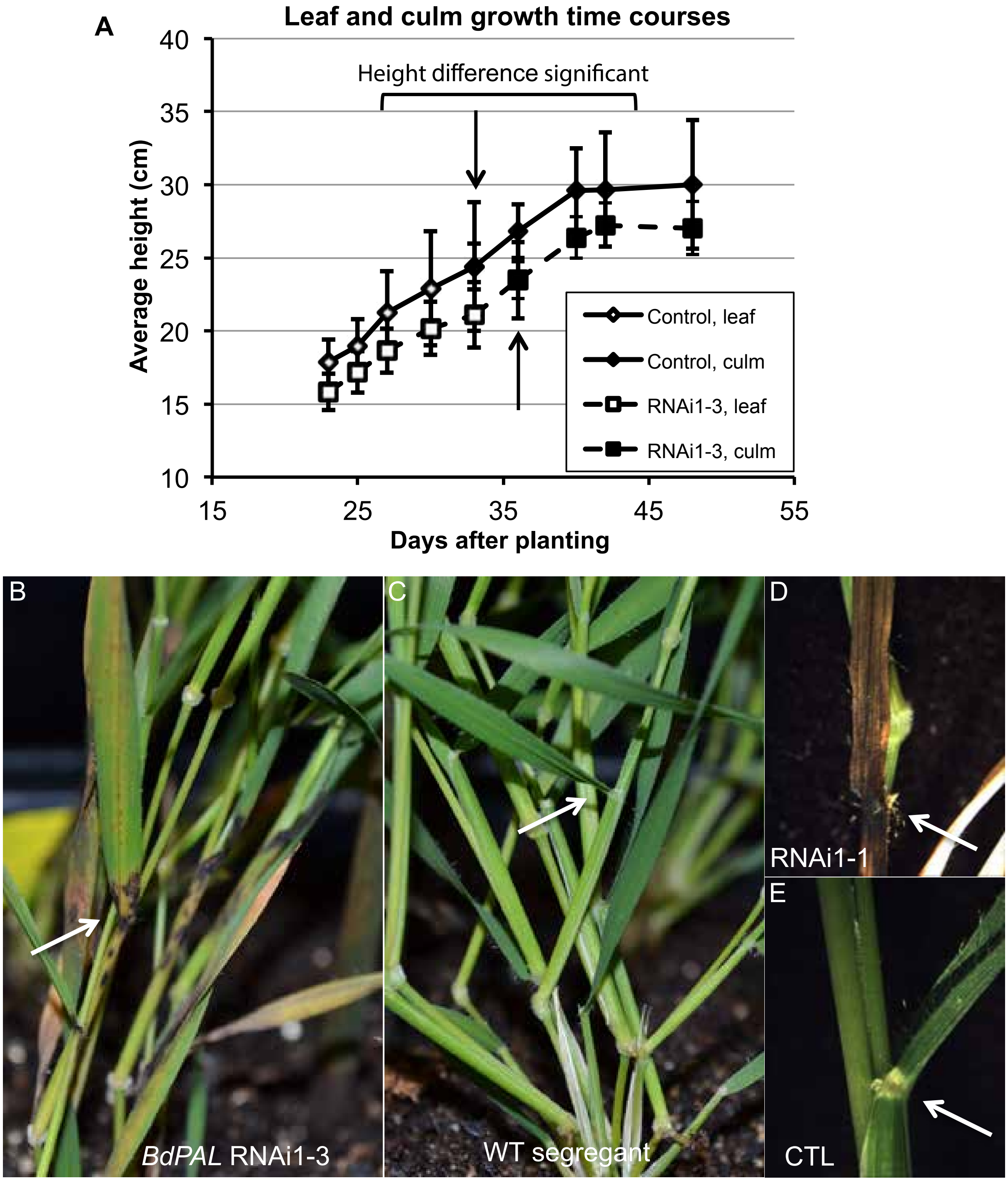

Figure S5. Growth time courses and phenotypes of *BdPAL* RNAi1 plants compared to CTL. **(A)** Time course measurements of *BdPAL* RNAi1-3 plants' longest leaves and tallest culms. Arrows delineate the time points at which 50% of spikelets could be seen emerging from the leaf whorl. The bracket delineates time points for which mutant average culm heights were significantly different from CTL (Student's t-test, $p < 0.05$). Bars represent SD. *BdPAL* RNAi1-3 **(B)** and RNAi1-1 **(D)** plants, at times, developed blackened spots or zones at the leaf collars (arrow), which may be due to fungal infections. CTL plants **(C, E)** never developed these phenotypes under the same growth chamber conditions.

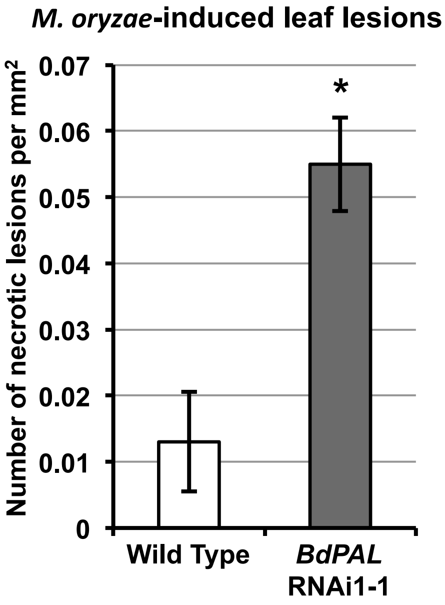

Figure S6. Effect of *BdPAL* silencing on *Magnaporthe oryzae* disease resistance. Values represent the average number of necrotic lesions per mm^2 of leaf surface, 5 days after spray inoculation. Bars represent standard errors (SE). Asterisk represents Student's t-test p-value < 0.001 comparing *BdPAL* RNAi1-1 to WT. 27 < n < 30.

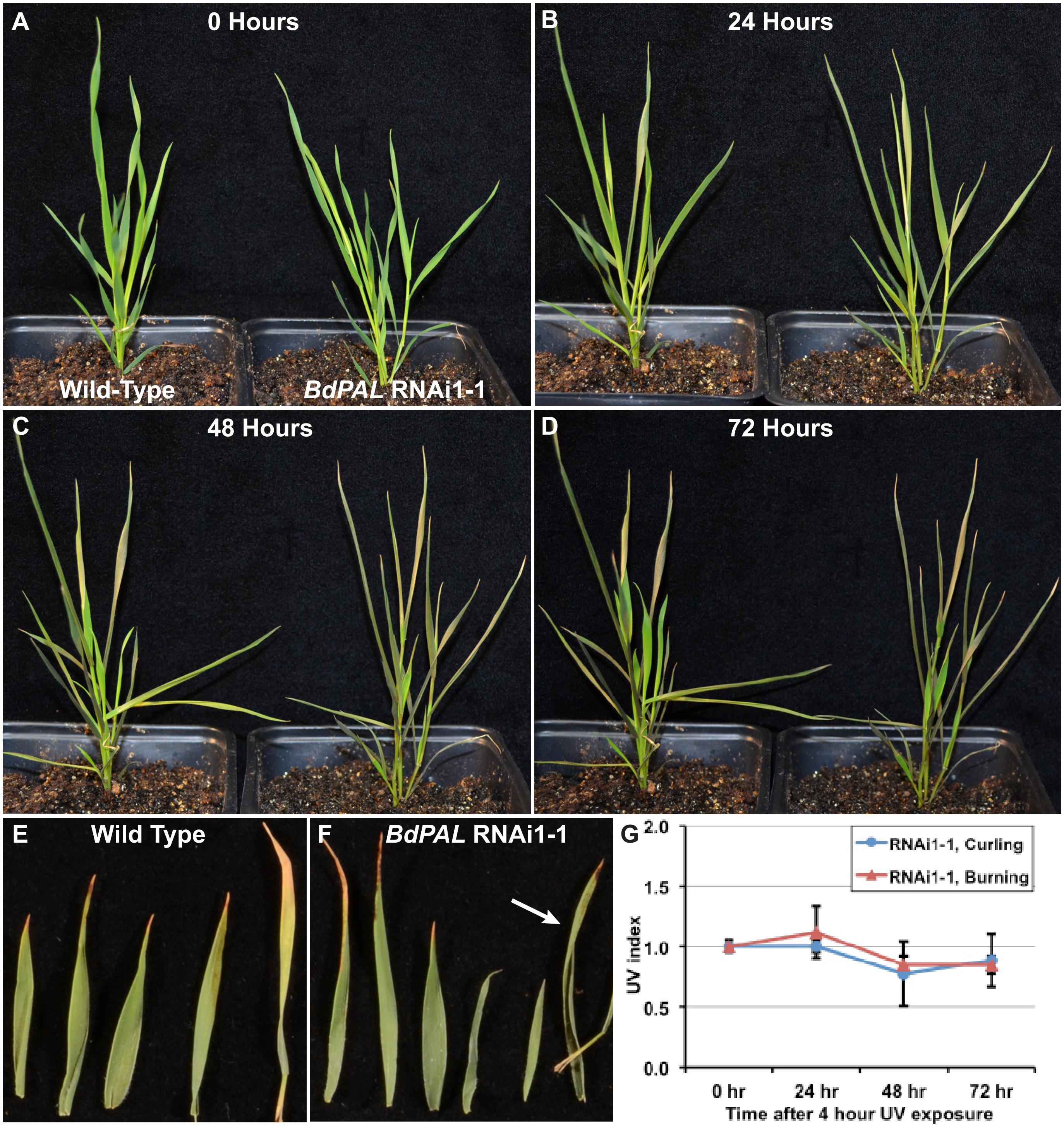

Figure S7. WT and *BdPAL* RNAi1-1 plant growth responses to 4 hours of high intensity UV-B light treatment. The plant on the left in **(A)** through **(D)** is WT whereas the plant on the right is *BdPAL* RNAi1-1. Note that both plants, which are representative of the population (n=15), developed relatively the same amounts of leaf browning and curling at the same rates. **(E)** and **(F)** Close-up images of representative leaves 48 hours after treatment (arrow delineates a leaf blade with UV-B damage-induced curling). **(G)** Quantification of leaf damage following UV-B light exposure, using a derived variable "UV index" for either curling or burned leaves for each plant. The UV index was calculated as [(number RNAi1-1 leaves with UV damage + 1) / (total RNAi1-1 leaves + 1)] / [(number WT leaves with UV damage + 1) / total WT leaves + 1)]. Plants were randomly assigned to pairs. UV index for each WT plant = 1. UV means were compared using Student's T-test, with no significant differences between RNAi1-1 and WT identified. Error bars represent Standard Deviation (SD).

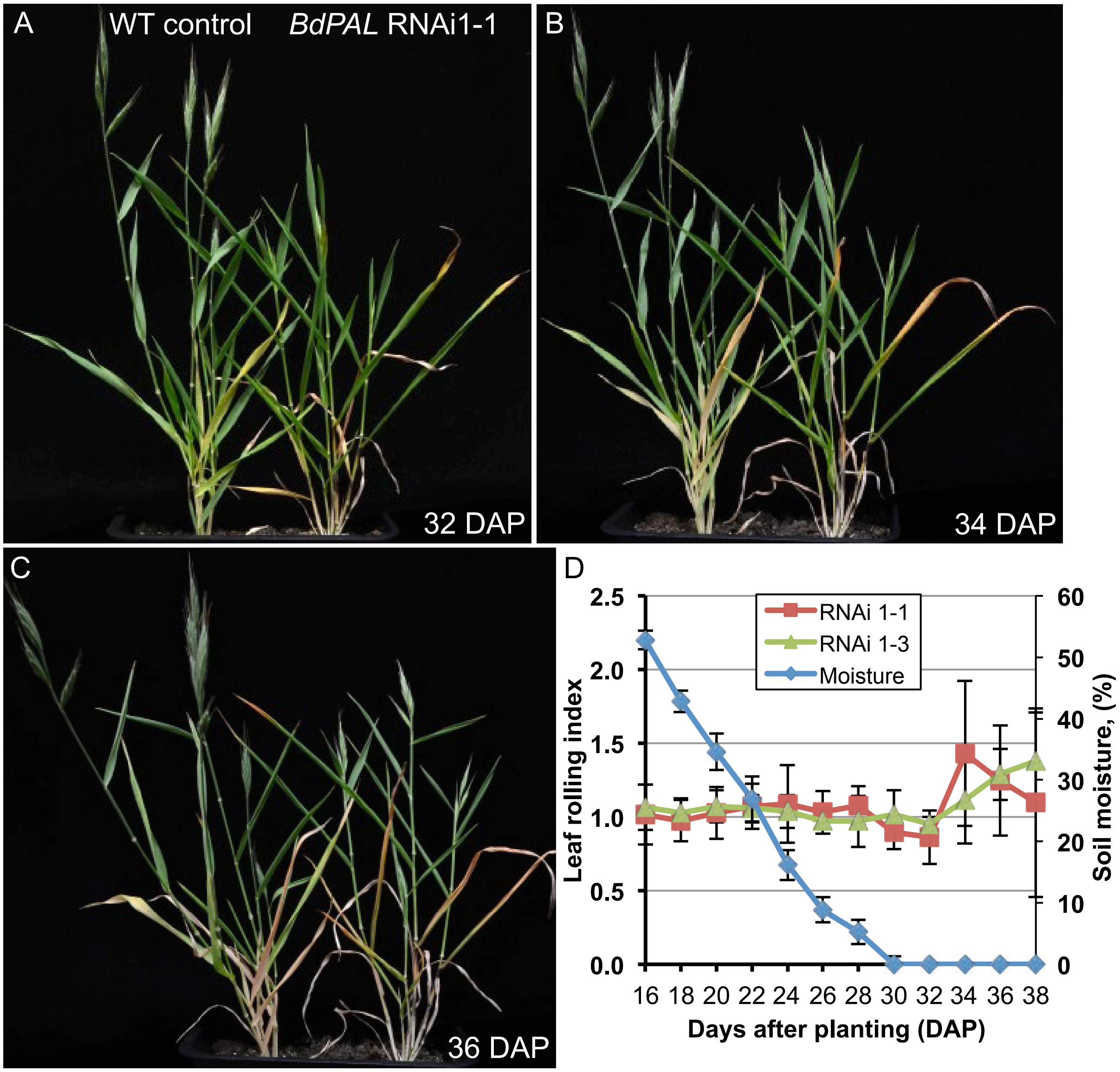

Figure S8. WT empty vector control (left) and *BdPAL* RNAi1-1 (right) plant growth responses to drought. Shown are the same two plants 32 **(A)**, 34 **(B)**, and 36 **(C)** days after planting (DAP). Watering was stopped on day 16, and the first signs of drought stress (leaf curling) were visible on day 33. **(E)** Leaf rolling as a response to drought was quantified using a derived variable “Leaf rolling index” for each pot. The leaf rolling index was calculated as [(number rolled leaves by genotype + 1) / (total number leaves by genotype + 1)] / [(number WT empty vector control rolled leaves + 1) / (total number WT empty vector leaves + 1)]. The leaf rolling index means were compared using Student’s T-test. A Bonferroni correction was applied to control for Type I error (α' = 0.0018). No significant differences were observed between each mutant line and WT at each time point. Error bars are SD.

Table S1. Genes predicted by PlaNet to be co-expressed with Bradi3g49250 (*BdPAL1*). http://aranet.mpimp-golm.mpg.de/index.html Known and predicted monolignol biosynthetic genes are marked in yellow.

**Gene ID**	**Description (most are putative, all are expressed)**	**Name**
Bradi5g25090.1	IQ calmodulin_binding motif family protein	
Bradi2g34240.1	CESA1 _ cellulose synthase	CESA1
Bradi2g55340.1	transmembrane amino acid transporter protein	
Bradi2g26760.1	annexin	
Bradi2g48267.1	alliin lyase precursor	
Bradi3g13237.1	transferase family protein	
Bradi2g54680.1	laccase precursor protein	
Bradi2g17067.1	auxin_responsive protein	
Bradi1g01870.1	pirin	
Bradi2g10970.1	tubulin/FtsZ domain containing protein	
Bradi3g11470.4	ras_related protein	
Bradi1g45487.1	annexin	
Bradi5g10017.1	FAD binding domain containing protein	
Bradi1g67870.1	expressed protein	
Bradi1g13910.1	START domain containing protein	
Bradi3g16670.1	peptide transporter PTR2	
Bradi5g20130.1	MYB family transcription factor	
Bradi3g39170.1	harpin_induced protein 1 domain containing protein	
Bradi4g24650.1	abscisic stress_ripening	
Bradi3g56020.1	aquaporin protein	
Bradi4g44530.1	peroxidase precursor	
Bradi2g59400.1	exostosin family domain containing protein	
Bradi2g10820.1	BRASSINOSTEROID INSENSITIVE 1_associated receptor kinase 1 precursor	
Bradi3g03460.1	flavonol sulfotransferase	
Bradi1g67460.1	phospholipase A2	
Bradi1g14050.1	uncharacterized protein At4g06744 precursor	
Bradi1g09460.1	endoglucanase	
Bradi1g25117.1	CSLF2 _ cellulose synthase_like family F; beta1,3;1,4 glucan synthase	
Bradi4g01200.2	5_methyltetrahydropteroyltriglutamate__homocysteine methyltransferase	
Bradi1g06090.1	ubiquitin_conjugating enzyme	
Bradi5g15490.1	ARPC2B	
Bradi1g54250.1	CESA8 _ cellulose synthase	CESA8
Bradi2g46197.1	no apical meristem protein	
Bradi1g32920.1	nodulin MtN3 family protein	
Bradi3g51387.1	aquaporin protein	
Bradi1g19660.1	uncharacterized GPI_anchored protein At5g19240 precursor	
Bradi4g22250.1	dirigent	
Bradi1g50050.1	lung seven transmembrane domain containing protein	
Bradi2g48280.1	systemin receptor SR160 precursor	
Bradi3g48530.1	transferase family protein	HCT
Bradi1g72762.1	hydrolase, alpha/beta fold family protein	
Bradi3g47950.1	expressed protein	
Bradi2g22170.1	expressed protein	
Bradi3g32180.1	expressed protein	
Bradi3g37530.1	ferric reductase	
Bradi1g28490.1	fiber protein Fb34	
Bradi2g60310.1	zinc finger protein	
Bradi4g31130.1	ferric reductase	
Bradi2g27060.1	golgi transport complex protein_related	
Bradi3g36887.1	cinnamoyl_CoA reductase	CCR
Bradi2g08790.1	Cupin domain containing protein	

Bradi5g14720.1	transferase family protein	HCT
Bradi2g21860.1	NADH_cytochrome b5 reductase	
Bradi1g03880.1	AP2 domain containing protein	
Bradi1g31320.1	AMP_binding domain containing protein	4CL
Bradi1g26510.1	glucan endo_1,3_beta_glucosidase precursor	
Bradi3g05750.1	AMP_binding domain containing protein	4CL
Bradi1g35477.1	STRUBBELIG_RECEPTOR FAMILY 7 precursor	
Bradi2g21300.1	cytochrome P450	C3'H
Bradi1g33230.1	expressed protein	
Bradi1g45710.1	plastocyanin_like domain containing protein	
Bradi1g21410.1	expressed protein	
Bradi1g06800.1	tubulin/FtsZ domain containing protein	
Bradi2g17982.1	myb_like DNA_binding domain containing protein	
Bradi3g05010.1	tubulin/FtsZ domain containing protein	
Bradi2g55730.1	cytochrome P450	
Bradi3g16530.1	O_methyltransferase	COMT
Bradi1g03947.1	leaf senescence related protein	
Bradi1g71680.2	LTPL69 _ Protease inhibitor/seed storage/LTP family protein precursor	
Bradi5g19960.1	RING_H2 finger protein ATL5G	
Bradi1g14110.1	expressed protein	
Bradi2g46810.1	expressed protein	
Bradi1g64830.1	glycosyl transferase 8 domain containing protein	
Bradi1g54280.1	expressed protein	
Bradi3g36217.1	endonuclease/exonuclease/phosphatase family domain containing protein	
Bradi4g21790.1	peptide transporter PTR2	
Bradi1g68280.1	actin_depolymerizing factor	
Bradi4g28260.1	hydroxyproline_rich glycoprotein family protein	
Bradi2g61070.1	NADPH quinone oxidoreductase	
Bradi1g65750.1	glycosyltransferase family 43 protein	
Bradi4g44860.1	PMR5	
Bradi2g26770.1	annexin	
Bradi5g02460.1	cytochrome P450 93A2	
Bradi1g03940.1	leaf senescence related protein	
Bradi2g37970.1	glycosyltransferase family 43 protein	
Bradi1g29560.1	pollen signalling protein with adenylyl cyclase activity	
Bradi2g23370.1	laccase precursor protein	
Bradi1g32850.1	RIC10	
Bradi2g08350.1	receptor_like protein kinase 5 precursor	
Bradi3g13420.1	esterase	
Bradi1g60750.1	phospho_2_dehydro_3_deoxyheptonate aldolase, chloroplast precursor	
Bradi2g12150.2	S_adenosylmethionine synthetase	
Bradi3g28350.1	CESA7 _ cellulose synthase	CESA7
Bradi1g59820.1	legume lectins beta domain containing protein	
Bradi4g21240.1	plant_specific domain TIGR01627 family protein	
Bradi1g75940.1	protein phosphatase 2C	
Bradi4g21220.1	auxin_induced protein 5NG4	
Bradi2g59410.1	exostosin family domain containing protein	
Bradi2g05927.1	harpin_induced protein 1 domain containing protein	
Bradi1g26110.1	expressed protein	
Bradi1g33160.1	auxin response factor 18	
Bradi1g06290.1	fasciclin domain containing protein	
Bradi4g13670.1	patatin	
Bradi1g72430.1	STRUBBELIG_RECEPTOR FAMILY 6 precursor	
Bradi3g49260.1	phenylalanine ammonia_lyase	PAL
Bradi1g58997.1	peroxidase precursor	

Bradi5g12460.1	uncharacterized Cys_rich domain containing protein	
Bradi3g54370.1	heparanase_like protein precursor	
Bradi4g40570.1	amino acid transporter	
Bradi4g37490.1	OsFBK20 _ F_box domain and kelch repeat containing protein	
Bradi3g59810.1	endoglucanase	
Bradi3g58730.1	dehydration response related protein	
Bradi1g64650.1	expressed protein	
Bradi1g00710.1	expressed protein	
Bradi3g45160.1	harpin_induced protein 1 domain containing protein	
Bradi1g34670.1	glycosyltransferase	
Bradi4g01717.1	galactosyltransferase family protein	
Bradi1g13680.1	nuclear transcription factor Y subunit	
Bradi1g65530.1	expressed protein	
Bradi1g60852.1	expressed protein	
Bradi3g52790.1	basic helix_loop_helix	
Bradi3g38950.1	methyladenine glycosylase	
Bradi2g12370.1	GDSL_like lipase/acylhydrolase	
Bradi1g06560.1	glycosyltransferase protein	
Bradi2g47590.1	MYB family transcription factor	
Bradi3g00377.1	rhodanese_like domain containing protein	
Bradi2g54940.1	homeodomain protein	
Bradi2g00220.1	fasciclin domain containing protein	
Bradi2g56970.1	amino acid transporter	
Bradi2g34470.1	solute carrier family 35 member E3	
Bradi3g30670.1	dehydration response related protein	
Bradi4g35477.1	bile acid sodium symporter family protein	
Bradi2g43690.1	oxidoreductase, aldo/keto reductase family protein	
Bradi2g49912.1	CESA4 _ cellulose synthase	CESA4
Bradi4g34300.1	membrane protein	
Bradi1g50280.1	expressed protein	
Bradi4g30540.1	CESA9 _ cellulose synthase	CESA9
Bradi1g11090.1	GRAS family transcription factor domain containing protein	
Bradi3g06480.1	dehydrogenase	CAD
Bradi5g10210.1	OsSub41 _ Putative Subtilisin homologue	
Bradi2g40437.1	GLUCAN SYNTHASE_LIKE protein	
Bradi2g36910.1	transferase family protein	PMT
Bradi1g68040.1	UDP_glucuronate 4_epimerase	
Bradi4g34040.1	CHIT13 _ Chitinase family protein precursor	
Bradi2g18447.1	sulfotransferase domain containing protein	
Bradi1g34790.1	arogenate dehydrogenase 1, chloroplast precursor	
Bradi1g47767.1	inorganic H+ pyrophosphatase	
Bradi1g72350.1	glycosyl transferase	
Bradi1g17830.1	potassium transporter	
Bradi3g40850.1	ras_related protein	
Bradi3g49250.2	phenylalanine ammonia_lyase	PAL
Bradi5g25767.1	auxin response factor	
Bradi4g01370.1	DNA binding protein	
Bradi3g04920.1	auxin response factor 6	
Bradi2g23530.1	homeodomain protein	
Bradi4g16560.1	cytochrome P450	C3'H
Bradi1g66720.1	laccase precursor protein	
Bradi2g56397.1	proton_dependent oligopeptide transport	
Bradi4g04420.1	boron transporter protein	
Bradi4g36240.1	endoglucanase	
Bradi2g52470.1	ICE_like protease p20 domain containing protein	

Bradi3g34615.2	OsSCP22 _ Putative Serine Carboxypeptidase homologue
Bradi3g05670.1	STRUBBELIG_RECEPTOR FAMILY 3 precursor
Bradi2g16560.1	fasciclin domain containing protein
Bradi2g51990.1	bHelix_loop_helix transcription factor
Bradi5g08907.1	heparan_alpha_glucosaminide N_acetyltransferase
Bradi4g40400.1	plant_specific domain TIGR01627 family protein
Bradi3g33070.1	expressed protein
Bradi1g10150.1	tubulin/FtsZ domain containing protein
Bradi3g58560.1	plastocyanin_like domain containing protein

**Mapman termID**	**Term description**	**Terms in NVN**	**Terms on microarray**	***p*-value**
10	cell wall	11	390	0
10.1.6	cell wall.precursor synthesis.GAE	1	5	0.03
10.2	cell wall.cellulose synthesis	6	47	0
10.2.1	cell wall.cellulose synthesis.cellulose synthase	6	23	0
10.5	cell wall.cell wall proteins	3	53	0.01
10.5.1	cell wall.cell wall proteins.AGPs	2	19	0
10.5.1.1	cell wall.cell wall proteins.AGPs.AGP	2	19	0.01
11.9.3.4	lipid metabolism.lipid degradation.lysophospholipases.phospholipase A2	1	4	0.05
12.1.1	N-metabolism.nitrate metabolism.NR	1	10	0.03
13.1	amino acid metabolism.synthesis	4	170	0.04
13.1.3	amino acid metabolism.synthesis.aspartate family	2	48	0.05
13.1.3.4	amino acid metabolism.synthesis.aspartate family.methionine	2	23	0
13.1.6.1.1	amino acid metabolism.synthesis.aromatic aa.chorismate.3-deoxy-D-arabino-heptulosonate 7-phosphate synthase	1	4	0.02
13.1.6.4	amino acid metabolism.synthesis.aromatic aa.tyrosine	1	3	0
13.1.6.4.1	amino acid metabolism.synthesis.aromatic aa.tyrosine.arogenate dehydrogenase \& prephenate dehydrogenase	1	3	0.02
16	secondary metabolism	13	350	0
16.1	secondary metabolism.simple phenols	3	35	0
16.2	secondary metabolism.phenylpropanoids	8	115	0
16.2.1	secondary metabolism.phenylpropanoids.lignin biosynthesis	5	39	0
16.2.1.1	secondary metabolism.phenylpropanoids.lignin biosynthesis.PAL	2	8	0
16.2.1.3	secondary metabolism.phenylpropanoids.lignin biosynthesis.4CL	2	16	0
16.5.99	secondary metabolism.sulfur-containing.misc	1	3	0
16.5.99.1	secondary metabolism.sulfur-containing.misc.alliinase	1	3	0.01
17.2.3	hormone metabolism.auxin.induced-regulated-responsive-activated	3	92	0.02
20.1.1	stress.biotic.respiratory burst	2	9	0
26	misc	18	1538	0.03
26.19	misc.plastocyanin-like	2	49	0.02
26.3.4	misc.gluco-, galacto- and mannosidases.endoglucanase	2	17	0.01
27.3.14	RNA.regulation of transcription.CCAAT box binding factor family, HAP2	1	5	0.02
27.3.22	RNA.regulation of transcription.HB,Homeobox transcription factor family	3	75	0
27.3.25	RNA.regulation of transcription.MYB domain transcription factor family	3	114	0.02
27.3.4	RNA.regulation of transcription.ARF, Auxin Response Factor family	3	21	0
29.7	protein.glycosylation	3	69	0.01
3.6	minor CHO metabolism.callose	1	13	0.05
31	cell	11	651	0
31.1	cell.organisation	10	352	0
34	transport	12	1010	0.04
34.13	transport.peptides and oligopeptides	3	108	0.03
34.19	transport.Major Intrinsic Proteins	2	30	0.02
34.19.1	transport.Major Intrinsic Proteins.PIP	2	11	0
34.3	transport.amino acids	4	85	0

Table S2. Genes predicted by PlaNet to be co-expressed with Bradi3g49260 (*BdPAL2*). http://aranet.mpimp-golm.mpg.de/index.html Known and predicted monolignol biosynthetic genes are marked in yellow.

Gene ID	Description (most are putative, all are expressed)	Name
Bradi5g25090.1	IQ calmodulin_binding motif family protein	
Bradi2g55340.1	transmembrane amino acid transporter protein	
Bradi1g75220.1	Citrate transporter protein	
Bradi2g26760.1	annexin	
Bradi1g58380.1	ERD4 protein	
Bradi3g37650.1	12_oxophytodienoate reductase	
Bradi3g13237.1	transferase family protein	
Bradi2g54680.1	laccase precursor protein	
Bradi2g17067.1	auxin_responsive protein	
Bradi1g01870.1	pirin	
Bradi2g10970.1	tubulin/FtsZ domain containing protein	
Bradi1g45487.1	annexin	
Bradi1g21490.1	zinc_finger protein	
Bradi3g16670.1	peptide transporter PTR2	
Bradi5g20130.1	MYB family transcription factor	
Bradi4g32190.1	EF hand family protein	
Bradi3g39170.1	harpin_induced protein 1 domain containing protei	
Bradi4g24650.1	abscisic stress_ripening	
Bradi4g44530.1	peroxidase precursor	
Bradi1g04140.1	methylenetetrahydrofolate reductase	
Bradi2g59400.1	exostosin family domain containing protein	
Bradi1g31820.1	vacuolar_sorting receptor precursor	
Bradi3g54320.1	expressed protein	
Bradi1g67460.1	phospholipase A2	
Bradi1g68040.1	UDP_glucuronate 4_epimerase	
Bradi1g09460.1	endoglucanase	
Bradi1g25117.1	CSLF2 _ cellulose synthase_like family F; beta1,3;1,4 glucan synthase	
Bradi4g01200.2	5_methyltetrahydropteroyltriglutamate__homocysteine methyltransferase	
Bradi1g71680.2	LTPL69 _ Protease inhibitor/seed storage/LTP family protein precursor	
Bradi5g15490.1	ARPC2B	
Bradi2g46197.1	no apical meristem protein	
Bradi1g32920.1	nodulin MtN3 family protein	
Bradi3g51387.1	aquaporin protein	
Bradi4g22250.1	dirigent	
Bradi1g50050.1	lung seven transmembrane domain containing protein	
Bradi3g48530.1	transferase family protein	HCT
Bradi3g47950.1	expressed protein	
Bradi2g22170.1	expressed protein	
Bradi3g32180.1	expressed protein	
Bradi3g37530.1	ferric reductase	
Bradi1g28490.1	fiber protein Fb34	
Bradi1g66620.1	clathrin assembly protein	
Bradi4g31130.1	ferric reductase	
Bradi3g36887.1	cinnamoyl_CoA reductase	CCR
Bradi2g08790.1	Cupin domain containing protein	
Bradi5g14720.1	transferase family protein	HCT
Bradi3g55840.1	Citrate transporter protein	
Bradi5g12460.1	uncharacterized Cys_rich domain containing protein	
Bradi1g31320.1	AMP_binding domain containing protein	4CL

Bradi3g54387.1	monogalactosyldiacylglycerol synthase	
Bradi3g05750.1	AMP_binding domain containing protein	4CL
Bradi2g34470.1	solute carrier family 35 member E3	
Bradi2g21300.1	cytochrome P450	C3'H
Bradi1g74420.1	sulfate transporter	
Bradi1g08120.1	UDP_glucose 6_dehydrogenase	
Bradi1g33230.1	expressed protein	
Bradi1g21410.1	expressed protein	
Bradi1g06800.1	tubulin/FtsZ domain containing protein	
Bradi2g17982.1	myb_like DNA_binding domain containing protein	
Bradi3g01080.1	tetratricopeptide repeat domain containing protein	
Bradi2g39420.1	ABC transporter, ATP_binding protein	
Bradi3g05010.1	tubulin/FtsZ domain containing protein	
Bradi2g55730.1	cytochrome P450	
Bradi3g16530.1	O_methyltransferase	COMT
Bradi1g14050.1	uncharacterized protein At4g06744 precursor	
Bradi1g06090.1	ubiquitin_conjugating enzyme	
Bradi5g19960.1	RING_H2 finger protein ATL5G	
Bradi2g46810.1	expressed protein	
Bradi3g36217.1	endonuclease/exonuclease/phosphatase family domain containing protein	
Bradi4g21790.1	peptide transporter PTR2	
Bradi1g68280.1	actin_depolymerizing factor	
Bradi4g28260.1	hydroxyproline_rich glycoprotein family protein	
Bradi2g61070.1	NADPH quinone oxidoreductase	
Bradi1g65750.1	glycosyltransferase family 43 protein	
Bradi4g44860.1	PMR5	
Bradi2g26770.1	annexin	
Bradi5g02460.1	cytochrome P450 93A2	
Bradi1g03940.1	leaf senescence related protein	
Bradi4g41990.1	harpin_induced protein 1 domain containing protein	
Bradi2g37970.1	glycosyltransferase family 43 protein	
Bradi1g13290.1	5_methyltetrahydropteroyltriglutamate__homocysteine methyltransferase	
Bradi1g29560.1	pollen signalling protein with adenylyl cyclase activity	
Bradi2g23370.1	laccase precursor protein	
Bradi1g32850.1	RIC10	
Bradi3g13420.1	esterase	
Bradi1g60750.1	phospho_2_dehydro_3_deoxyheptonate aldolase, chloroplast precursor	
Bradi2g12150.2	S_adenosylmethionine synthetase	
Bradi3g28350.1	CESA7 _ cellulose synthase	CESA7
Bradi1g75940.1	protein phosphatase 2C	
Bradi4g21220.1	auxin_induced protein 5NG4	
Bradi2g59410.1	exostosin family domain containing protein	
Bradi4g08917.1	BTBN18 _ Bric_a_Brac, Tramtrack, Broad Complex BTB domain with non_phototropic hypocotyl 3 NPH3 domain	
Bradi1g20080.1	sec20 domain containing protein	
Bradi1g26110.1	expressed protein	
Bradi3g29970.1	hydrolase, alpha/beta fold family domain containing protein	
Bradi1g33160.1	auxin response factor 18	
Bradi1g06290.1	fasciclin domain containing protein	
Bradi4g13670.1	patatin	
Bradi1g72430.1	STRUBBELIG_RECEPTOR FAMILY 6 precursor	
Bradi3g49260.1	phenylalanine ammonia_lyase	PAL
Bradi1g75730.1	cytochrome P450 86A1	
Bradi1g68750.1	transmembrane 9 superfamily member	

Bradi1g58997.1	peroxidase precursor	
Bradi3g31440.1	hydrolase, alpha/beta fold family protein	
Bradi4g40570.1	amino acid transporter	
Bradi5g19037.1	rhoGAP domain containing protein	
Bradi4g37490.1	OsFBK20 _ F_box domain and kelch repeat containing protein	
Bradi3g59810.1	endoglucanase	
Bradi1g00710.1	expressed protein	
Bradi3g45160.1	harpin_induced protein 1 domain containing protein	
Bradi2g37950.1	tetraspanin family protein	
Bradi1g34670.1	glycosyltransferase	
Bradi1g54680.1	vesicle_associated membrane protein	
Bradi1g13680.1	nuclear transcription factor Y subunit	
Bradi1g36950.1	myristoyl_acyl carrier protein thioesterase, chloroplast precursor	
Bradi1g65530.1	expressed protein	
Bradi1g60852.1	expressed protein	
Bradi3g52790.1	basic helix_loop_helix	
Bradi3g38950.1	methyladenine glycosylase	
Bradi3g50930.1	scarecrow	
Bradi2g12370.1	GDSL_like lipase/acylhydrolase	
Bradi2g47590.1	MYB family transcription factor	
Bradi3g00377.1	rhodanese_like domain containing protein	
Bradi3g23330.1	ras_related protein	
Bradi2g54940.1	homeodomain protein	
Bradi2g56970.1	amino acid transporter	
Bradi1g35477.1	STRUBBELIG_RECEPTOR FAMILY 7 precursor	
Bradi2g49912.1	CESA4 _ cellulose synthase	CESA4
Bradi4g34300.1	membrane protein	
Bradi1g50280.1	expressed protein	
Bradi4g30540.1	CESA9 _ cellulose synthase	CESA9
Bradi1g44510.1	B12D protein	
Bradi3g10120.1	expressed protein	
Bradi1g11090.1	GRAS family transcription factor domain containing protein	
Bradi3g06480.1	dehydrogenase	CAD
Bradi5g08907.1	heparan_alpha_glucosaminide N_acetyltransferase	
Bradi1g75410.1	N_acetylglucosaminyltransferase	
Bradi2g36910.1	transferase family protein	PMT
Bradi2g52470.1	ICE_like protease p20 domain containing protein	
Bradi4g34040.1	CHIT13 _ Chitinase family protein precursor	
Bradi3g33070.1	expressed protein	
Bradi1g72350.1	glycosyl transferase	
Bradi1g17830.1	potassium transporter	
Bradi3g49250.2	phenylalanine ammonia_lyase	PAL
Bradi2g27060.1	golgi transport complex protein_related	
Bradi4g01370.1	DNA binding protein	
Bradi3g04920.1	auxin response factor 6	
Bradi2g23530.1	homeodomain protein	
Bradi4g16560.1	cytochrome P450	C3'H
Bradi1g67100.1	OsWLIM2 _ LIM domain protein, putative actin_binding protein and transcription factor	
Bradi1g66720.1	laccase precursor protein	
Bradi4g03680.1	heparanase_like protein precursor	
Bradi2g58630.2	BHLH transcription factor	
Bradi2g56397.1	proton_dependent oligopeptide transport	
Bradi4g04420.1	boron transporter protein	

Bradi4g36240.1	endoglucanase
Bradi2g35440.1	lipase
Bradi5g25860.1	OsRR6 type_A response regulator
Bradi2g16560.1	fasciclin domain containing protein
Bradi3g58560.1	plastocyanin_like domain containing protein
Bradi5g10210.1	OsSub41 _ Putative Subtilisin homologue
Bradi4g40400.1	plant_specific domain TIGR01627 family protein
Bradi1g47767.1	inorganic H+ pyrophosphatase
Bradi1g10150.1	tubulin/FtsZ domain containing protein
Bradi3g36460.1	RAD23 DNA repair protein
Bradi4g35477.1	bile acid sodium symporter family protein

**Mapman termID**	**Term description**	**Terms in NVN**	**Terms on microarray**	***p*-value**
10	cell wall	9	390	0
10.1.4	cell wall.precursor synthesis.UGD	1	4	0.05
10.1.6	cell wall.precursor synthesis.GAE	1	5	0.02
10.2	cell wall.cellulose synthesis	4	47	0
10.2.1	cell wall.cellulose synthesis.cellulose synthase	4	23	0
10.5	cell wall.cell wall proteins	2	53	0.01
11.1.7	lipid metabolism.FA synthesis and FA elongation.ACP thioesterase	1	5	0.03
11.10.1	lipid metabolism.glycolipid synthesis.MGDG synthase	1	3	0.01
11.9.3.4	lipid metabolism.lipid degradation.lysophospholipases.phospholipase A2	1	4	0.02
13	amino acid metabolism	4	232	0.05
13.1	amino acid metabolism.synthesis	4	170	0.01
13.1.3	amino acid metabolism.synthesis.aspartate family	3	48	0
13.1.3.4	amino acid metabolism.synthesis.aspartate family.methionine	3	23	0
13.1.6.1.1	amino acid metabolism.synthesis.aromatic aa.chorismate.3-deoxy-D-arabino-heptulosonate 7-phosphate synthase	1	4	0.03
16	secondary metabolism	12	350	0
16.1	secondary metabolism.simple phenols	3	35	0
16.2	secondary metabolism.phenylpropanoids	8	115	0
16.2.1	secondary metabolism.phenylpropanoids.lignin biosynthesis	5	39	0
16.2.1.1	secondary metabolism.phenylpropanoids.lignin biosynthesis.PAL	2	8	0
16.2.1.10	secondary metabolism.phenylpropanoids.lignin biosynthesis.CAD	1	7	0
16.2.1.3	secondary metabolism.phenylpropanoids.lignin biosynthesis.4CL	2	16	0
17.7.1.5	hormone metabolism.jasmonate.synthesis-degradation.12-Oxo-PDA-reductase	1	11	0.02
20.1.1	stress.biotic.respiratory burst	2	9	0
26	misc	17	1538	0.05
26.3.4	misc.gluco-, galacto- and mannosidases.endoglucanase	2	17	0
27.3.14	RNA.regulation of transcription.CCAAT box binding factor family, HAP2	1	5	0.04
27.3.22	RNA.regulation of transcription.HB,Homeobox transcription factor family	2	75	0.05
27.3.25	RNA.regulation of transcription.MYB domain transcription factor family	3	114	0.03
27.3.4	RNA.regulation of transcription.ARF, Auxin Response Factor family	2	21	0.01
31	cell	13	651	0
31.1	cell.organisation	11	352	0
33	development	10	484	0
33.99	development.unspecified	9	409	0
34	transport	13	1010	0.03
34.13	transport.peptides and oligopeptides	3	108	0
34.3	transport.amino acids	4	85	0

Table S3. Lignin compositional analyses for Brachypodium wild type (WT), *BdPAL* RNAi1-1, empty vector control (CTL), and *BdPAL* RNAi1-3 senesced stem plus leaf sheath tissue. Ferulate (FA) measured by the mild alkaline hydrolysis assay was released from cell wall hemicelluloses, whereas *p*-coumarate (*p*CA) was released from both lignin and hemicelluloses.

Genotype/ phenotype	Klason lignin content (%)			Relative frequency of lignin-derived thioacidolysis monomers				Mild alkaline hydrolysis	
	Acid-insoluble	Acid-soluble	Total	% H	%G	%S	S/G ratio	FA (µg/mg)	*p*CA (µg/mg)
WT	15.3 ± 0.2	3.8 ± 0.1	19.1 ± 0.2	1.7 ± 0.15	33.2 ± 1.6	65.0 ± 1.6	2.0 ± 0.2	6.7 ± 0.6	6.9 ± 0.4
RNAi1-1	7.7 ± 0.7*	3.0 ± 0.2*	10.8 ± 1.0*	5.0 ± 0.96*	20.2 ± 1.8*	74.8 ± 1.0*	3.7 ± 0.4*	2.9 ± 0.3*	5.8 ± 0.1*
CTL	17.8 ± 0.2	2.9 ± 0.1	20.7 ± 0.2	2.0 ± 0.05	41.0 ± 1.4	56.9 ± 1.3	1.4 ± 0.1	6.0 ± 0.3	7.5 ± 0.1
RNAi1-3	11.3 ± 0.3*	3.1 ± 0.0*	14.4 ± 0.3*	4.1 ± 0.21*	25.3 ± 1.0*	70.6 ± 0.8*	2.8 ± 0.1*	6.1 ± 0.2	6.7 ± 0.6

WT and RNAi1-1 plants were grown together and therefore their related values were compared to each other, as were CTL and RNAi1-3. Values are means ± Standard Deviation (SD) from three independent pools of tissue (three technical reps for each). Asterisks denote Student's t-test significant differences ($p<0.006$).

Table S4. Comparison of RNA-seq derived average transcript levels of putative *CESA* genes in *BdPAL* RNAi1-1 stem plus leaf sheath tissues versus empty vector control (CTL).

**Locus I.D.**	**Gene I.D.**	**Gene Name**	**CTL**	***BdPAL* RNAi1-1**	**Fold Change**	***p*val**	***p*adj**
LOC100821105	Bradi2g34240	BdCESA1	8414	7815	0.9	0.457	1
LOC100822586	Bradi1g04597	BdCESA2	568	550	1.0	0.778	1
LOC100833611	Bradi1g54250	BdCESA3	9444	9298	1.0	0.907	1
LOC100830844	Bradi3g28350	BdCESA4*	11717	11371	1.0	0.710	1
LOC100832508	Bradi1g29060	BdCESA5	413	335	0.8	0.133	1
LOC100838115	Bradi1g53207	BdCESA6	2890	2695	0.9	0.422	1
LOC100843814	Bradi4g30540	BdCESA7*	17379	16137	0.9	0.382	1
LOC100837994	Bradi2g49912	BdCESA8*	23474	21141	0.9	0.271	1

*BdCESA4, 7, and 8 are thought to carry out secondary cell wall cellulose biosynthesis (Handakumbura et al., 2013). *p*val and *p*adj stand for *p*-value and adjusted *p*-value, respectively. n=3.

Table S5. Comparison of RNA-seq derived average transcript levels of putative monolignol biosynthetic genes in *BdPAL* RNAi1-1 stem plus leaf sheath tissues versus CTL. The first ten genes were predicted by PlaNet to be co-expressed with *BdPAL1* and *BdPAL2*, whereas the bottom three genes were identified as encoding proteins having the highest amino acid sequence identities with other known proteins in lignin biosynthetic pathways.

Locus I.D.	Gene I.D.	Gene Name	CTL	*BdPAL* RNAi1-1	Fold Change	*p*val	*p*adj
LOC100842862	Bradi3g05750	4CL	6381	9159	1.4	0.160	1
LOC100842528	Bradi1g31320	4CL	1312	1315	1.0	0.872	1
LOC100840920	Bradi2g36910	PMT[1]	2716	5003	1.8	0.011	0.474
LOC100823638	Bradi5g14720	HCT	2620	2836	1.1	0.449	1
LOC100821527	Bradi3g48530	HCT	1890	2666	1.4	0.584	1
LOC100835531	Bradi2g21300	C3'H	2648	3691	1.4	0.0003	0.050
LOC100844617	Bradi4g16560	C3'H	10496	13090	1.2	0.445	1
LOC100838607	Bradi3g16530	COMT[2]	30049	41795	1.4	0.230	1
LOC100832790	Bradi3g36887	CCR[3]	3902	6991	1.8	0.184	1
LOC100843666	Bradi3g06480	CAD[4]	6811	11702	1.7	0.019	0.638
LOC100839986	Bradi2g53470	C4H	3316	4883	1.5	0.043	0.925
LOC100835440	Bradi3g39420	CCoAOMT	11918	16457	1.4	0.114	1
LOC100833404	Bradi3g30590	F5H	1441	2844	2.0	0.459	1

Upper case numbers next to gene names denote genes experimentally shown to be involved in lignin biosynthesis: [1]Petrik et al., 2014; [2] Dalmais et al., 2013; Trabucco et al., 2013; [3]Cass and Sedbrook, unpublished; [4]Bouvier d'Yvoire et al., 2013. *p*val and *p*adj stand for p-value and adjusted *p*-value, respectively. n=3 for RNAi1-1 and CTL samples, each.
